# Archaeal Host Cell Recognition and Viral Binding of HFTV1 to Its *Haloferax* Host

**DOI:** 10.1128/mbio.01833-22

**Published:** 2023-01-19

**Authors:** Sabine Schwarzer, Thomas Hackl, Hanna M. Oksanen, Tessa E. F. Quax

**Affiliations:** a Archaeal Virus-Host Interactions, Faculty of Biology, University of Freiburg, Freiburg, Germany; b Groningen Institute for Evolutionary Life Sciences, University of Groningen, Groningen, The Netherlands; c Molecular and Integrative Biosciences Research Programme, Faculty of Biological and Environmental Sciences, University of Helsinki, Helsinki, Finland; d Biology of Archaea and Viruses, Groningen Biomolecular Sciences and Biotechnology Institute, University of Groningen, Groningen, The Netherlands; University of Vienna

**Keywords:** *Haloarchaea*, *Caudoviricetes*, archaeal virus, viral adsorption, infection mechanism, *Haloferax*, *Archaea*, viral entry

## Abstract

Viruses are highly abundant and the main predator of microorganisms. Microorganisms of each domain of life are infected by dedicated viruses. Viruses infecting archaea are genomically and structurally highly diverse. Archaea are undersampled for viruses in comparison with bacteria and eukaryotes. Consequently, the infection mechanisms of archaeal viruses are largely unknown, and most available knowledge stems from viruses infecting a select group of archaea, such as crenarchaea. We employed Haloferax tailed virus 1 (HFTV1) and its host, Haloferax gibbonsii LR2-5, to study viral infection in euryarchaea. We found that HFTV1, which has a siphovirus morphology, is virulent, and interestingly, viral particles adsorb to their host several orders of magnitude faster than most studied haloarchaeal viruses. As the binding site for infection, HFTV1 uses the cell wall component surface (S)-layer protein. Electron microscopy of infected cells revealed that viral particles often made direct contact with their heads to the cell surface, whereby the virion tails were perpendicular to the surface. This seemingly unfavorable orientation for genome delivery might represent a first reversible contact between virus and cell and could enhance viral adsorption rates. In a next irreversible step, the virion tail is orientated toward the cell surface for genome delivery. With these findings, we uncover parallels between entry mechanisms of archaeal viruses and those of bacterial jumbo phages and bacterial gene transfer agents.

## INTRODUCTION

Archaeal viruses represent the most unexplored part of the virosphere (1, 2). Archaea are ubiquitous microorganisms that colonize very diverse parts of our planet. They make up a considerable part of the biodiversity in the oceans, play important roles in biochemical cycling, and can live in hot springs or hydrothermal vents with temperatures around the boiling point, and they also grow on human skin and are found in the human gut (3–5). Archaea are evolutionarily more closely related to eukaryotes than to bacteria, although they are prokaryotic. They have unique properties, such as the archaeal cell envelope composition, which consists of ether-linked lipids with a glycerol-1-phosphate backbone, in contrast to the ester-based lipids with a glycerol-3-phosphate backbone in bacteria and eukaryotes (6). Whereas bacteria are usually covered in a peptidoglycan layer of murein, archaea lack murein and are instead nearly always wrapped in a surface (S)-layer consisting of glycosylated protein (7). As a consequence, archaeal viruses face different challenges to enter a host cell from bacterial or eukaryotic viruses (8). Archaeal viruses are highly diverse both with regard to their sequences and their structures. Some archaeal viruses have unique shapes, such as that of a bottle, spindle, or spiral, while the others have morphologies that can also be found for viruses of bacteria and/or eukaryotes, such as tailless icosahedral or a head-tail morphology (9). As archaeal viruses are understudied, it is still an open question if entry and egress mechanisms are conserved within all archaeal viruses or if such mechanisms mimic those of known bacterial viruses. Specifically, the entry mechanism of archaeal viruses is not well understood (8, 10, 11). Only a handful of receptors have been identified, and they include the S-layer protein or adhesive pili that are presented at the cell surface (12–14). The limited available information stems mainly from viruses infecting members of the *Crenarchaeota*. The entry mechanisms of double-stranded DNA (dsDNA) tailed bacteriophages have been studied in detail, such that for multiple phages the receptors on the host surface have been identified, as well as the virion proteins that are essential for entry (15). Genome sequences of tailed dsDNA archaeal viruses are quite diverse. As a result, it is difficult to predict archaeal virus gene function based on bacteriophage genomics (16). Since the cell envelopes of archaea and bacteria also differ significantly, entry mechanisms used by bacteriophages might not function in archaea. Therefore, unraveling of entry mechanisms of archaeal viruses relies on experimental approaches.

We aimed to explore the entry mechanism of archaeal tailed double-stranded DNA viruses, which are the most numerous archaeal virus isolates known today (2, 16). For this, we selected the haloarchaeon Haloferax gibbonsii LR2-5 and Haloferax tailed virus 1 (HFTV1, family *Haloferuviridae*, order *Kirjokansivirales*, and class *Caudoviricetes*), which has a siphovirus-like morphotype with a head connected to a long noncontractile tail (16–18). This is the first and only available virus isolated from a *Haloferax* host, and it serves as a model for haloarchaeal virus-host studies. HFTV1 was isolated together with its host from the hypersaline Lake Retba in Senegal (17). The genome sequence of H. gibbonsii LR2-5 is 3.8 Mb and revealed that LR2-5 does not contain a CRISPR-Cas antiviral defense mechanism, which might explain why it is one of the few *Haloferax* strains susceptible to viral infection (18, 19). LR2-5 has the typical rod shape of haloarchaea and is motile in the early exponential phase, whereas cells round up and lose their motility in the stationary phase (18). HFTV1 specifically infects H. gibbonsii LR2-5, and closely related strains such as H. gibbonsii Ma2.38 and Haloferax volcanii H26 are not susceptible to HFTV1 (17, 18). In this study, we found that HFTV1 uses the highly abundant S-layer protein as binding site and that it absorbed unusually fast in comparison to other haloarchaeal viruses. Electron microscopy revealed that HFTV1 can bind either with the head or the tail to the cell surface.

## RESULTS

### Effect of salinity and high temperature on HFTV1 stability.

**FIG 1:**
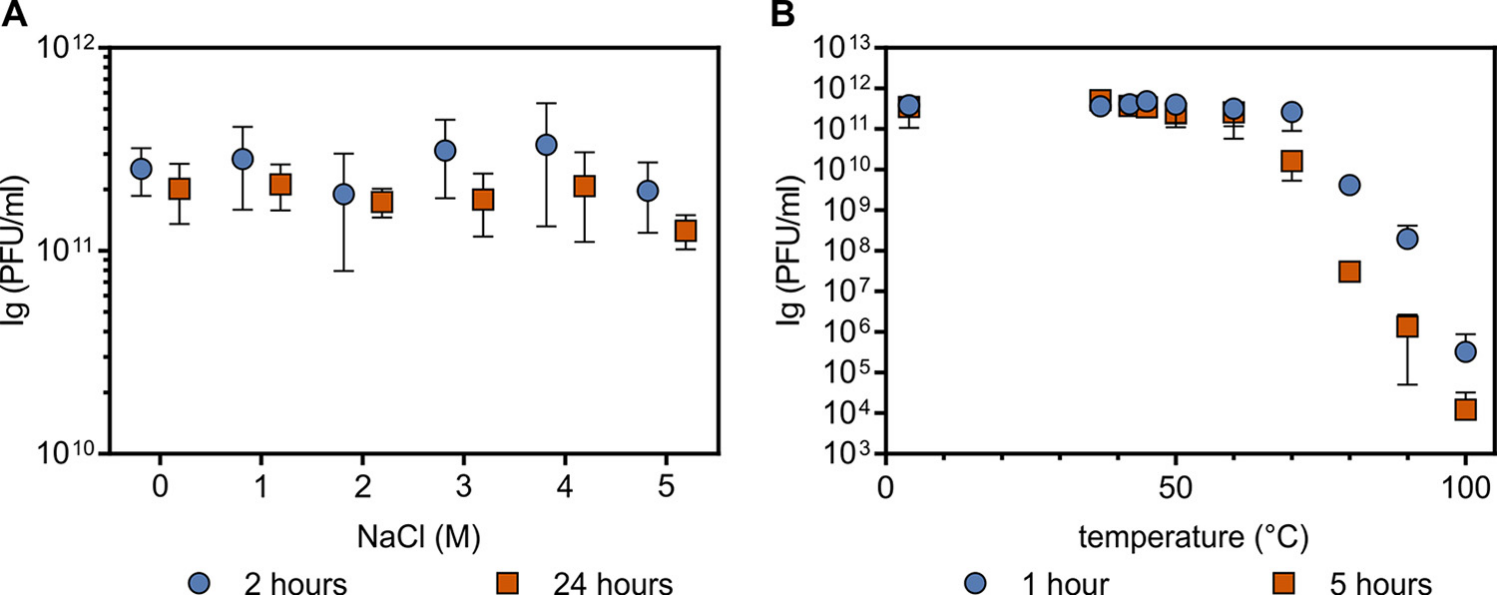
HFTV1 stability. (A) Infectivity at low and high NaCl concentrations. HFTV1 particles were diluted 1,000 times in MGM medium with different concentrations of NaCl and incubated for 2 or 24 h before the infectivity of the sample was determined by plaque assay. Error bars represent the standard deviation from three biological and technical replicates. (B) Temperature stability. The HFTV1 virus lysate was exposed to various temperatures for 1 h or 5 h. Subsequently, a plaque assay was performed to determine the number of infectious viral particles. Error bars represent the standard deviation from four independent experiments. If the error bars are not visible, the deviation could not be resolved graphically.

We tested the impact of various NaCl concentrations (0 to 5 M) on the stability of the HFTV1 particle and the efficiency of infection. HFTV1 particles were stable independent of NaCl concentrations (Fig. 1A). After 2 h, 3 × 10^11^ plaque-forming units (PFU)/mL were still detectable in 0 M NaCl, which was at the same level as the control in high salt. After 24 h, infectivity was 2 × 10^11^ PFU/mL. This shows that HFTV1 is very stable in low to almost saturated salt concentrations (4 to 5 M NaCl, also including 113 mM MgCl_2_, 108 mM MgSO_4_, and 71 mM KCl). Therefore, NaCl concentrations have no effect on infectivity. In general, the viral stock titer (on average, 5× 10^11^ PFU/mL) remained unchanged over a 6-month period (see Fig. S1 in the supplemental material).

10.1128/mbio.01833-22.2 FIG S1 HFTV1 is stable for at least 6 months at 4°C. The infectivity of HFTV1 agar stock was monitored over a 12-month period. The stock was stored at 4°C, and the titer (PFU/mL) was determined by plaque assay. Scale bars represent two technical replicates. Download FIG S1, TIF file, 0.5 MB. Copyright © 2023 Schwarzer et al. 2023 Schwarzer et al. https://creativecommons.org/licenses/by/4.0/ This content is distributed under the terms of the Creative Commons Attribution 4.0 International license.

After incubation of HFTV1 at various temperatures ranging from 50°C to 100°C, we observed that the infectivity remained unchanged up to 70°C. The infectivity decreased within 1 to 5 h when the temperature was higher than 80°C (Fig. 1B). At this temperature, the infectivity decreased 6 orders of magnitude but was not completely lost even at 100°C.

Thus, HFTV1 tolerates both large and small amounts of NaCl and temperatures up to 70°C, at least for relatively short time periods. These properties render the virus very robust to the changing environmental conditions in its natural habitat. This finding is in line with the previously detected wide global distribution of archaeal tailed viruses in different environments (16, 20). We decided to use a temperature of 37°C in further infection experiments.

### Virus infection leads to increase in host cell volume.

**FIG 2:**
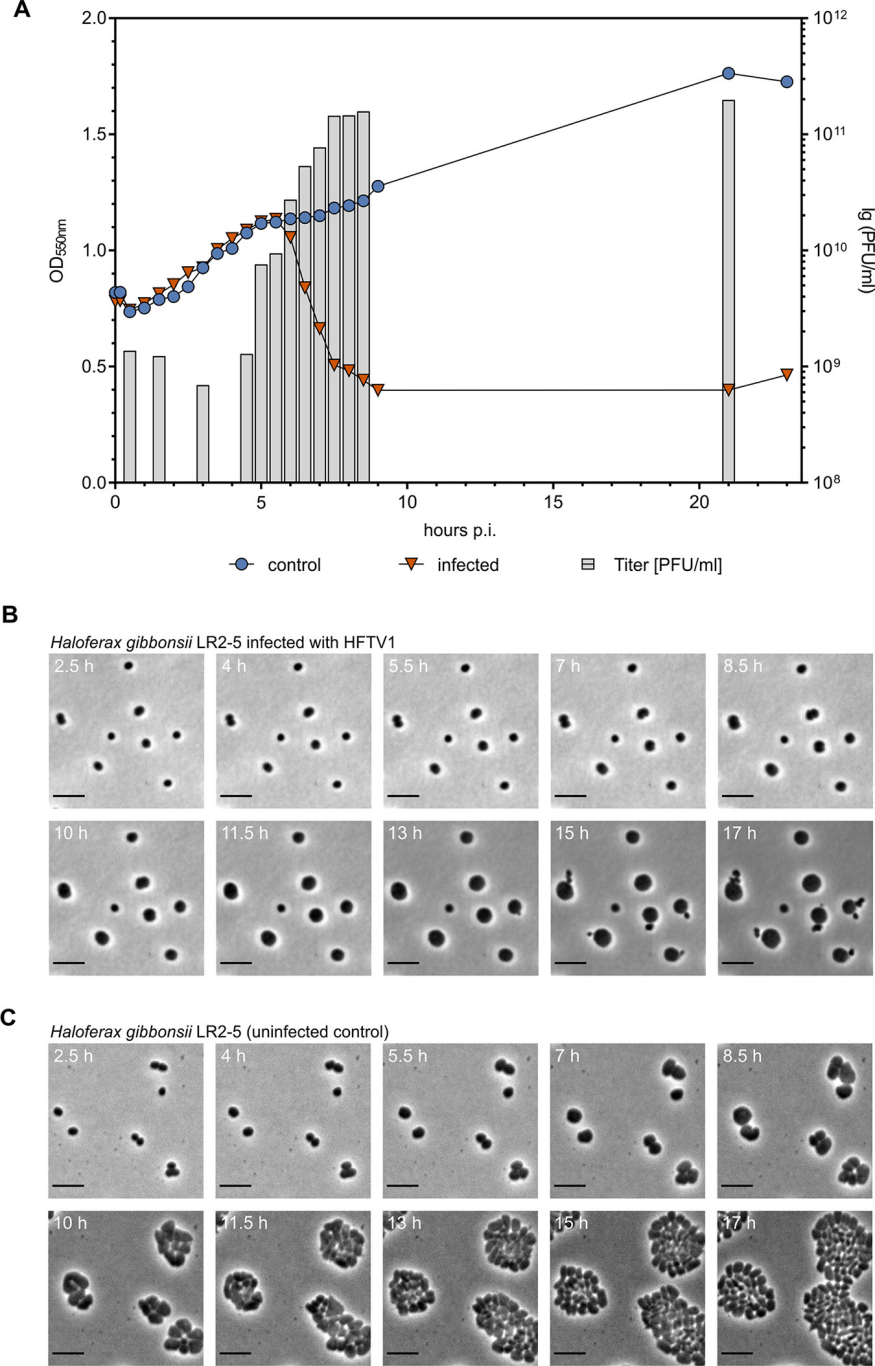
HFTV1 life cycle and time course of infection in Haloferax gibbonsii LR2-5. (A) Single-step growth curve of HFTV1. H. gibbonsii cells were grown to logarithmic phase (OD_550_ of 0.8, ~9.0 × 10^8^ CFU/mL) and infected at an MOI of 10 at 37°C. To remove unadsorbed viral particles, the cells were washed thoroughly 20 min postinfection and transferred to fresh medium at 37°C. The number of free viruses was monitored by plaque assay. (B) Time-lapse microscopy of H. gibbonsii cells during HFTV1 infection. (C) Time-lapse microscopy of uninfected control cells. Cells were grown on an agarose pad supplemented with Casamino Acids in a thermomicroscope set at 45°C. Selected phase-contrast images show cells at 2.5 to 17 hpi (1.5-h intervals) after infection with HFTV1 (B) or uninfected control cells (C). HFTV1-infected cells increase strongly in size, while they are not observed dividing. The first lysis of infected cells was observed between 11 and 13.5 hpi and is indicated by white arrows. The uninfected control cells started to divide after 8.5 to 11 h incubation. Scale bars, 5 μm.

We observed that HFTV1 forms clear plaques of 2 to 4 mm in diameter on host lawns of H. gibbonsii LR2-5, which is indicative of a lytic infection. This is similar to what is observed for other tailed haloarchaeal viruses releasing their progeny by host cell lysis (21). We determined the length of the infection cycle by performing a one-step growth experiment using a multiplicity of infection (MOI) of 10 (Fig. 2A) (Fig. S2). The extracellular virus titer rose after 6 h postinfection (hpi), indicating the length of the latency period. The infection resulted in a large number of progeny, and the average burst size was about 65 viruses per infected cell, resulting in typical titers of 7 × 10^10^ PFU/mL in the culture lysate. Around 6 hpi, also a drop in the optical density (OD) of the culture was measured. The number of viable cells was reduced over 3 orders of magnitude 21 hpi from 7 × 10^9^ CFU/mL to 2 × 10^6^ CFU/mL. Infected cells were followed by phase-contrast time-lapse microscopy (Movie S1), which revealed that cells first increase considerably in size (Fig. S3) before they finally burst at 11.5 to 13 hpi. Snapshots taken at different stages of the infection showed that infected cells (Fig. 2B), in contrast to noninfected cells (Fig. 2C), were no longer dividing (Movie S2). Their size doubled their volume such that cells immediately prior to virus release had diameters that were ~1.5-fold larger than control cells. Such an increase in cell volume has been observed previously for the crenarchaeal Sulfolobus tengchongensis spindle-shaped virus 2 (STSV2). However, in that case, the diameter increased even more to 20-fold that of control cells (22). Lysis of the LR2-5 cells in time lapse-microscopy was characterized by sudden leakage of cytoplasm in small burst events (Movie S1).

10.1128/mbio.01833-22.3 FIG S2 Single-step growth curves of HFTV1. H. gibbonsii LR2-5 cells were grown to logarithmic phase and infected at an MOI of 10 at 37°C. (A to C) Cultures infected at OD_600_ of 0.3 (~1.0 × 10^7^ CFU/mL) (A), OD_600_ of 0.8 (~2.0 × 10^9^ CFU/mL) (B), and OD_600_ of (~4.0 × 10^9^ CFU/mL) (C). To remove unadsorbed viral particles, the cells were washed thoroughly 20 min postinfection and transferred to fresh medium at 37°C. The growth curves of infected (triangles) and uninfected (circles) cultures and the number of free viruses (PFU/mL) are shown. Download FIG S2, TIF file, 1.8 MB. Copyright © 2023 Schwarzer et al. 2023 Schwarzer et al. https://creativecommons.org/licenses/by/4.0/ This content is distributed under the terms of the Creative Commons Attribution 4.0 International license.

10.1128/mbio.01833-22.4 FIG S3 Cell size increases during the time course of infection. Box plots showing cell area distributions of H. gibbonsii LR2-5 cells infected with HFTV1. Boxes represent values from >600 cells analyzed at 1 to 10 hpi (1.5-h intervals). After 10 hpi, cell area increased ~1.5-fold. A line within the box marks the median. The upper boundary represents the 75th percentile. The bottom whisker represents minimum values, and the top whisker represents maximum values. Download FIG S3, TIF file, 0.8 MB. Copyright © 2023 Schwarzer et al. 2023 Schwarzer et al. https://creativecommons.org/licenses/by/4.0/ This content is distributed under the terms of the Creative Commons Attribution 4.0 International license.

10.1128/mbio.01833-22.10 MOVIE S1 Phase-contrast time-lapse imaging of Haloferax gibbonsii LR2-5 infected with HFTV1. Cells from early log growth phase (OD_600_ of 0.2) were infected with HFTV1 at an MOI of 10. Cells were grown on an agarose nutrition pad in a thermomicroscope at 45°C and imaged every 10 minutes. Time postinfection is indicated in hours. Download Movie S1, AVI file, 14.8 MB. Copyright © 2023 Schwarzer et al. 2023 Schwarzer et al. https://creativecommons.org/licenses/by/4.0/ This content is distributed under the terms of the Creative Commons Attribution 4.0 International license.

10.1128/mbio.01833-22.11 MOVIE S2 Phase-contrast time-lapse imaging of noninfected Haloferax gibbonsii LR2-5 cells. Cells were grown on an agarose nutrition pad in a thermomicroscope at 45°C and imaged every 15 minutes. Time postinfection is indicated in hours. Download Movie S2, MOV file, 17.6 MB. Copyright © 2023 Schwarzer et al. 2023 Schwarzer et al. https://creativecommons.org/licenses/by/4.0/ This content is distributed under the terms of the Creative Commons Attribution 4.0 International license.

### HFTV1 is a fast-adsorbing virus, and its receptor is highly abundant.

**FIG 3:**
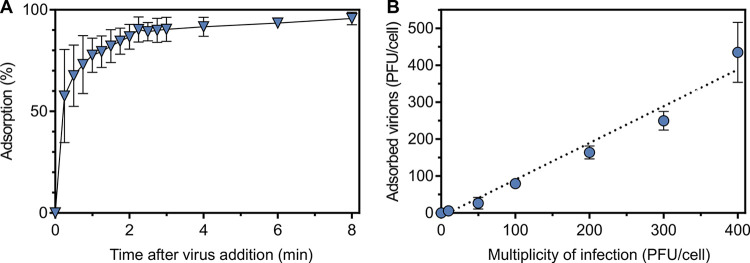
Adsorption efficiency of HFTV1 to H. gibbonsii cells. (A) To determine the adsorption rate of HFTV1, H. gibbonsii LR2-5 cells were grown to the mid-logarithmic phase (OD_600_ of 1.0; ~2 × 10^9^ CFU/mL) and infected with HFTV1 using an MOI of 0.001 at 37°C. The number of unbound virus particles was determined after 0 to 8 min postinfection by plaque assay. Error bars represent standard deviation from three experiments. (B) To determine if the receptor can be saturated, a constant number of H. gibbonsii LR2-5 cells (OD_600_ of 0.65 to 0.85, ~1 × 10^8^ to 6 × 10^8^ CFU/mL) were infected with HFTV1 at various MOIs ranging from 0.001 to 400. The number of unadsorbed particles present in the supernatant was determined by plaque assay 30 min postinfection and compared to the number of particles in a cell-free control. Error bars represent standard deviation from three independent experiments. If the error bars are not visible, the deviation could not be resolved graphically.

We used the so-called “inverted” viral adsorption assay to measure the rate by which particles attach to the cell surface, which relies on the measurement of the decrease of viral particles in the media after “pulsing” the cells with viruses (23). The adsorption assay showed that the binding of HFTV1 to host cells is extremely efficient and synchronized. Saturated adsorption occurs within the first 3 min after infection, resulting in 90% of the virions being bound to the host cells (Fig. 3A). The adsorption rate constant calculated at 10 min postinfection was 1.9 × 10^−9 ^mL min^−1^.

The rate of attachment of HFTV1 particles was not affected by preinfection with HFTV1 (Fig. S7A), indicating that superinfection exclusion of HFTV1 against subsequent infecting HFTV1 particles is not occurring.

10.1128/mbio.01833-22.8 FIG S7 Superinfection exclusion assay. H. gibbonsii LR2-5 cells were infected at an MOI of 10 and incubated for 1.5 h at 37°C. Unbound particles were removed by washing, and the cells were subjected to a second round of infection with HFTV1 at an MOI of 0.1. Blue circles show the adsorption kinetics of particles to uninfected control cells. Orange squares represent the adsorption to cells preinfected with HFTV1. Error bars represent the standard deviation of three independent experiments. If the error bars are not visible, the deviation could not be resolved graphically. Download FIG S7, TIF file, 0.6 MB. Copyright © 2023 Schwarzer et al. 2023 Schwarzer et al. https://creativecommons.org/licenses/by/4.0/ This content is distributed under the terms of the Creative Commons Attribution 4.0 International license.

To determine whether the receptor of HFTV1 is abundant or rarely exposed on the host cell surface (24), we performed receptor saturation experiments by infecting H. gibbonsii LR2-5 cells with HFTV1 at different MOI values from 0.001 to 400. Subsequently, the number of free particles remaining in the supernatant was determined and compared with the number of virions added to the cell-free control (Fig. 3B). Even samples infected with an MOI of 400 showed that about 30 min postinfection, ~400 viruses were bound to the cells, and saturation was observed. This indicates that the receptor mediating the primary interaction between HFTV1 and H. gibbonsii is very abundant.

### HFTV1 binds to cells with an unusual orientation with a tail pointing outward from the cells.

**FIG 4:**
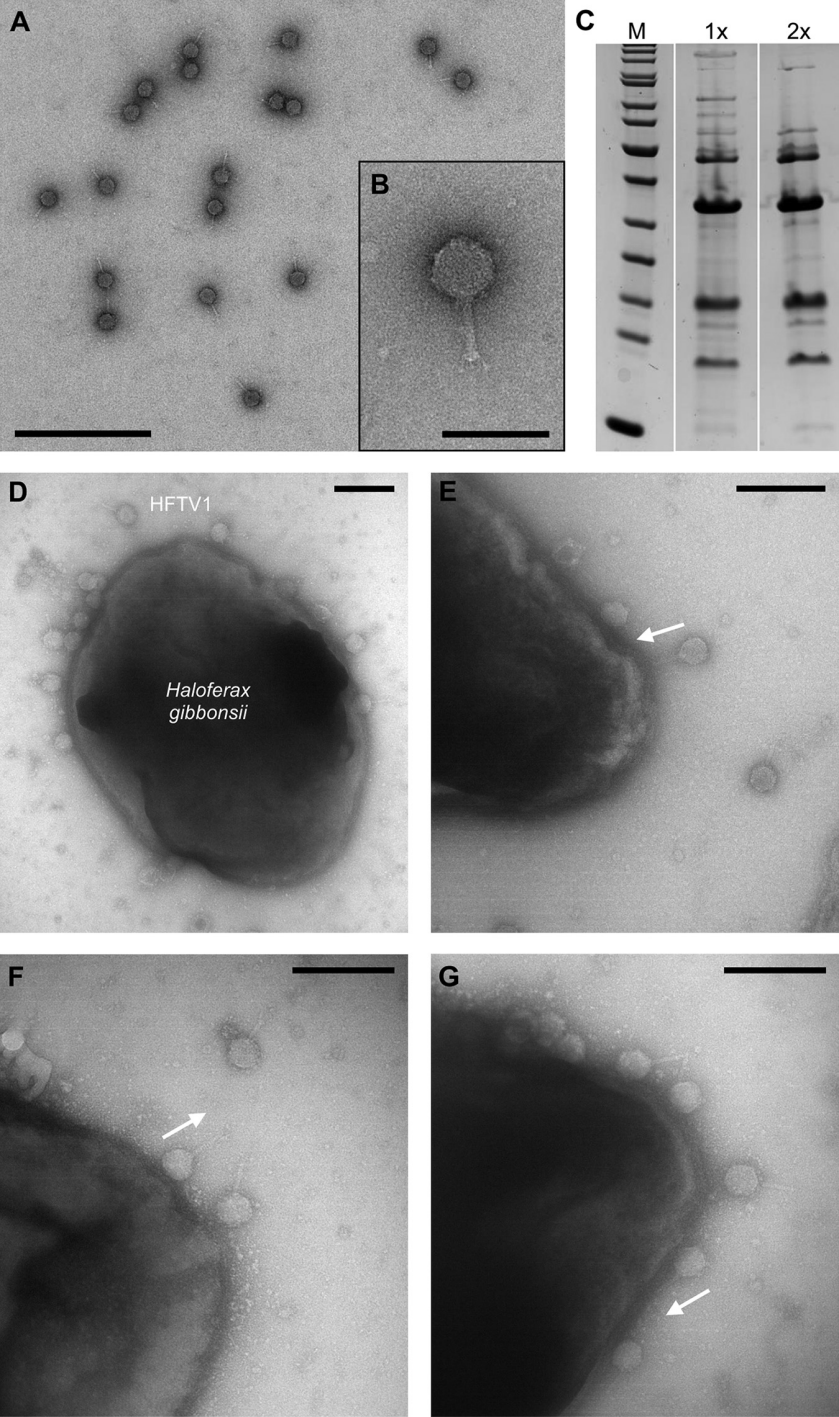
TEM of HFTV1 purification and particle binding to Haloferax gibbonsii. (A) Transmission electron microscopy of negatively stained 2× purified HFTV1. Scale bar, 500 nm. (B) Inset to panel A. Scale bar, 100 nm. (C) Major protein bands of 1× (purified by precipitation and rate zonal ultracentrifugation in sucrose) and 2× (purified by precipitation, rate zonal ultracentrifugation in sucrose, and equilibrium ultracentrifugation in CsCl) purified HFTV1 particles. Molecular mass marker (M) ranges from 200 kDa to 10 kDa (Thermo Scientific; catalog no. 26614). (D to G) Adsorption of HFTV1 to Haloferax gibbonsii LR2-5 cells. Scale bars, 200 nm. (D) TEM observation showing that particles attach with random orientations. Particles were observed binding with their tails toward the cell surface (E), in parallel to the surface (F), or with their heads (G). The orientations of tails of HFTV1 are indicated with white arrows pointing toward the tail tip. The majority (50 to 70%) of HFTV1 particles were examined attaching to the cell surface head side.

In order to observe the binding process of HFTV1 by electron microscopy, highly pure and infectious viral particles were produced. Purification of HFTV1 virions was optimized by changing the sucrose gradient from 5 to 20% (wt/vol) (17) to 10 to 40% (wt/vol) and using optimized time to separate viruses by rate-zonal centrifugation. The purification of polyethylene glycol-NaCl-precipitated viruses in a linear 10 to 40% (wt/vol) sucrose gradient resulted in two blue and one gray light-scattering band (Fig. S4A) that were separated from most of other sample components absorbing at 280 nm (Fig. S4B). Most of the infectivity was found in the lower blue band (a total of 80% of infectivity in three peak fractions [Fig. S4C]), resulting in high specific infectivity of ~1 × 10^13^ PFU/mg protein (Fig. S4D). Purifying the viruses further by equilibrium centrifugation in CsCl resulted in a single sharp light-scattering band and occasionally also a minor upper band with low infectivity. Recovery of the total amount of infectious viruses was >10% compared to that of the lysate, and the yield of the CsCl-purified viruses was 1 to 2 mg per L of lysate (*n* = 3). The specific infectivity of CsCl-purified virus was ~1 × 10^13^ to 3 × 10^13^ PFU/mg protein (*n* = 3). The transmission electron microscopy (TEM) and SDS-PAGE analysis of the protein profiles of the purified viruses confirmed the purity of the sample (Fig. 4A to C). TEM analysis showed that the purified HFTV1 sample was very homogeneous and devoid of impurities such as archaella or cell debris (Fig. 4A and B). In addition, the majority of the particles showed DNA-filled heads (Fig. 4A), in contrast to the majority of DNA-devoid particles that were obtained with the original purification method (17). It is noteworthy that this method separates the infectious viruses efficiently and produces significantly better-quality material with specific infectivity that was 4 orders of magnitude higher than previously reported (17), allowing the particles to be used in infection experiments.

10.1128/mbio.01833-22.5 FIG S4 Improved purification of HFTV1 particles by rate zonal centrifugation in sucrose. (A) Polyethylene glycol-NaCl-precipitated HFTV1 particles were separated in a linear 10 to 40% (wt/vol) sucrose gradient (18% SW buffer; 210,000 × *g*, 1.5 h, 15°C; Sorvall TH641 rotor) and fractionated into 12 fractions (numbering shown on left). (B to E) Fractions were analyzed by their absorbance at 280 nm (B), infectivity (total PFU) (C), specific infectivity (PFU/*A*_280_) (D), and protein content by Coomassie blue-stained SDS-PAGE gel (E). Fraction numbers are shown on top. The mass marker (in kilodaltons) is on left. Download FIG S4, TIF file, 0.2 MB. Copyright © 2023 Schwarzer et al. 2023 Schwarzer et al. https://creativecommons.org/licenses/by/4.0/ This content is distributed under the terms of the Creative Commons Attribution 4.0 International license.

To visualize the binding of HFTV1 to the host cell surface, we used TEM to observe negatively stained H. gibbonsii LR2-5 cells mixed with HFTV1 at different MOIs. Directly after adding the virus to the host cell, viral binding was observed. Numerous particles with full heads were visible at the cell surface of H. gibbonsii LR2-5 (Fig. 4D). HFTV1 bound to the cell all over the surface and did not accumulate at specific sites (Fig. 4D). Curiously, the largest fraction (50 to 70%) of the viral particles was found bound directly with the DNA-filled head to the cell surface. Their tails were parallel or even perpendicular to the host cell surface (Fig. 4E to G). This observation is in contrast to the visualized binding events of most tailed phages, which are typically orientated with the tail and tail fibers toward the cell surface.

### Escape mutants implicate one of two S-layer proteins as the primary receptor.

**FIG 5:**
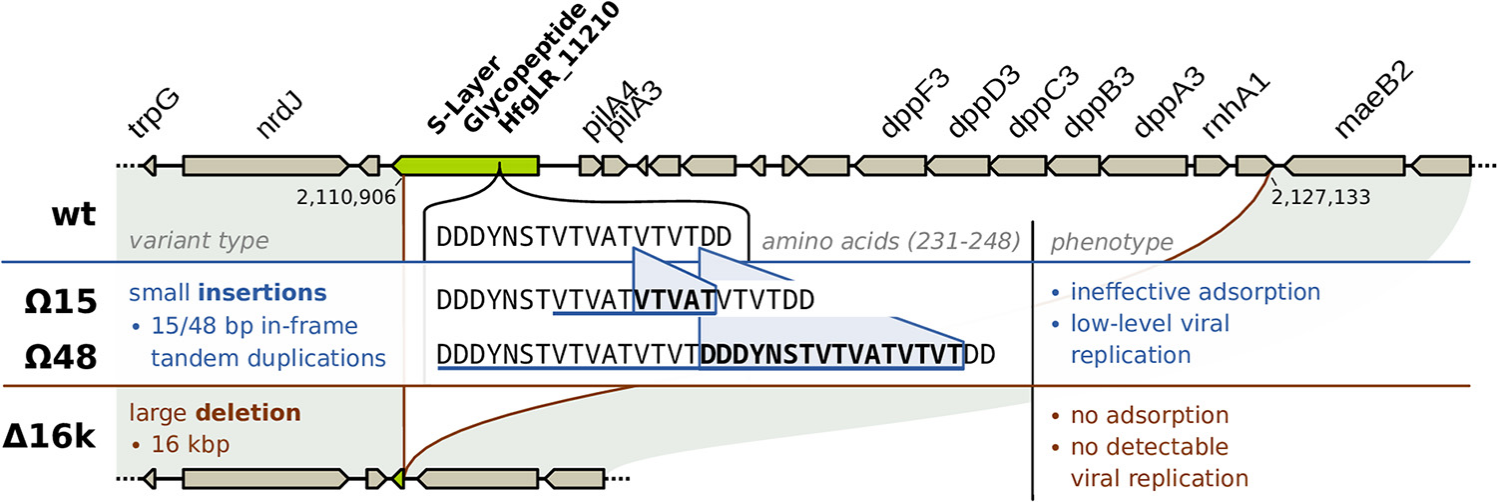
Sequence changes in H. gibbonsii LR2-5 mutants causing resistance to HFTV1 infection. Shown is a 25-kb region on the H. gibbonsii LR2-5 main chromosome for the wild type (wt, top) and escape mutants Ω15, Ω48, and Δ16k. Mutants Ω15 and Ω48 each carry a small insertion, 15 and 48 bases, respectively, in *hfgLR_11210*, which is one of two S-layer glycoproteins of H. gibbonsii LR2-5 (indicated in green). The changes at the protein level (amino acids in bold) caused by these tandem duplications are highlighted by blue triangles. Mutant Δ16k contains a 16.2-kb deletion affecting more than 90% of the coding region of *hfgLR_11210*.

To identify the HFTV1 receptor, we isolated “escape mutants” of the HFTV1 host strain, which are cells that survived a viral infection and were no longer susceptible to renewed HFTV1 infection. H. gibbonsii LR2-5 cells were challenged with HFTV1 in liquid culture, and the resulting lysate was plated to isolate resistant cells. Single colonies were colony purified, regrown, and rechallenged with HFTV1 by spot assay (Fig. S6). Three LR2-5 strains did not support HFTV1 plaque formation and were sent for whole-genome sequencing. Analysis of the sequences of three escape mutants, Ω48, Δ16K, and Ω15, revealed clear differences from the sequence of the wild-type HFTV1-susceptible strain (Fig. 5). All changes were found in the region around the gene *HfgLR_11210*, which encodes one of the two S-layer proteins of H. gibbonsii LR2-5. S-layer proteins are the major cell wall components of haloarchaea, and several haloarchaea, such as H. gibbonsii LR2-5, encode two different S-layer proteins (18). In the escape mutants Ω15 and Ω48, small 15-bp and 48-bp in-frame tandem duplications, respectively, had occurred in the gene *HfgLR_11210*. This region encodes a threonine-rich motif of the S-layer protein (237 to 246 amino acids). Mutant Δ16K has a large 16-kb deletion, affecting more than 90% of the *HfgLR_11210* gene and also several other adjacently located genes (Fig. 5). These include pilA3 and pilA4, as well as several currently nonannotated genes, followed by dppF3 and dppA3 encoding parts of an ABC transporter system, rnhA1, which encodes an RNase, and maeB2 (putative malate dehydrogenase). Analysis of the susceptibility of the three mutants to HFTV1 by spot assays showed that strains were able to grow in liquid medium (Fig. S5) and on plates in a layer of soft agar. Cells in a soft layer showed no lysis after infection with HFVTV1 (Fig. S6). There were reads mapping to the HFVTV1 sequence in Ω15 and Ω48 sequences, while these were completely absent in Δ16K (Table S1 in Text S1). *HfgLR_11210* encodes an S-layer glycopeptide, which is part of the cell wall of LR2-5 (18). We conclude that this S-layer glycopeptide is likely the receptor for HFTV1 and that the binding between HFTV1 and S-layer glycopeptide probably can be hampered by the small insertions around positions 237 to 246. We hypothesize that a very inefficient adsorption may still take place. These few adsorption events will still result in viral replication and are thus responsible for the detected HFTV1 reads in these mutants. In case of Δ16K, where almost the complete S-layer glycopeptide gene is deleted, presumably, no adsorption can take place, and thus, no HFTV1 reads are detected. This is also consistent with our observation that the binding to Δ16K was reduced to 50 to 60%, showing a significant effect of the S-layer glycoprotein gene deletion on adsorption of HFTV1 (Fig. S7B). Due to the low sequence similarity of *HfgLR_11210* to the S-layer of H. volcanii, of which the structure has been solved (25), it was not possible to predict if the small amino acid insertions were in a particular domain of the S-layer protein structure. The deletion of one of the S-layer proteins is not lethal for H. gibbonsii LR2-5, and the Δ16K escape mutants grow at the same rate as the original strain. We cannot exclude that under variable environmental conditions (i.e., low salinity, low nutrients, biofilm formation), there might be a fitness burden for this escape mutant with only one S-layer protein.

10.1128/mbio.01833-22.1 TEXT S1 Supplemental material and methods. Download Text S1, DOCX file, 0.04 MB. Copyright © 2023 Schwarzer et al. 2023 Schwarzer et al. https://creativecommons.org/licenses/by/4.0/ This content is distributed under the terms of the Creative Commons Attribution 4.0 International license.

10.1128/mbio.01833-22.6 FIG S5 Growth of H. gibbonsii LR2-5 and its escape mutants in liquid culture. (A) Typical growth curve of H. gibbonsii LR2-5, Ω15, Ω48, and Δ16k in MGM medium with 18% SW. The average optical density was calculated from three independent technical replicates. Error bars represent the standard deviation. (B) Typical growth curve of H. gibbonsii LR2-5, Ω15, Ω48, and Δ16k in CA medium with 18% SW. (C) Phase-contrast light microscopy images show the cell shapes of H. gibbonsii LR2-5 (OD_600_ of 0.45) and its escape mutants Ω15 (OD_600_ of 0.44), Ω48 (OD_600_ of 0.26), and Δ16k (OD_600_ of 0.31) at mid-exponential growth phases. Download FIG S5, TIF file, 2.8 MB. Copyright © 2023 Schwarzer et al. 2023 Schwarzer et al. https://creativecommons.org/licenses/by/4.0/ This content is distributed under the terms of the Creative Commons Attribution 4.0 International license.

10.1128/mbio.01833-22.7 FIG S6 Susceptibility of Haloferax gibbonsii LR2-5 and escape mutants to HFTV1. Spot-on-lawn assay conducted with lawns of H. gibbonsii LR2-5, Δ16K, Ω48, and Ω15. Different dilutions of HFTV1 lysate (undiluted 3 × 10^11^ PFU/mL) were spotted on the respective host lawns and incubated for 4 days. Clearing of the cellular lawn appeared only on H. gibbonsii LR2-5 lawns, whereas no indication of cell lysis was observed when HFTV1 lysate was spotted on the host lawns of the escape mutants Δ16K, Ω48, and Ω15. Download FIG S6, TIF file, 2.6 MB. Copyright © 2023 Schwarzer et al. 2023 Schwarzer et al. https://creativecommons.org/licenses/by/4.0/ This content is distributed under the terms of the Creative Commons Attribution 4.0 International license.

## DISCUSSION

*Haloferuviridae* is a small and diverse archaeal viral family belonging to the order *Caudoviricetes* (16). Haloferuvirus HFTV1 is the only virus isolate infecting a *Haloferax* host (17, 19), making it an interesting model to study the entry and infection mechanism of an archaeal virus in high detail.

HFTV1 particles can cope with temperatures between 4°C to 70°C, which is a much wider temperature distribution than the temperature from the isolation site in hypersaline Lake Retba, Senegal, where the average temperature is 25°C (26), but within the range of other viruses of haloarchaea (27, 28). HFTV1 was tolerant to a broad NaCl concentration surviving also at “zero salt.” It was reported before that tailed haloarchaeal viruses (non-lipid containing) are usually more resistant to changes in ionic strength, whereas membrane-containing viruses are more sensitive to changing NaCl concentrations (with enveloped viruses being the most sensitive) (29). Generally, haloarchaeal viruses can withstand a wider range of NaCl concentrations than their hosts (28, 29).

HFTV1 particles adsorb to the host cell within minutes. This makes HFTV1 several orders of magnitude faster than most haloarchaeal viruses studied (Table 1). So far, only one haloarchaeal virus, VOLN27B, is known to adsorb to its host *Halorubrum* sp. strain LN27 in less than a minute (27). The known adsorption rate constants of haloarchaeal viruses range from ~10^−10^ to 10^−13 ^mL min^−1^, in contrast to viruses of crenarchaea or bacteria that have adsorption rate constants of ~10^−9^ to 10^−10 ^mL min^−1^ (Table 1). The current hypothesis is that the high ionic strength under which haloarchaeal viruses infect might result in a natural slower adsorption than viruses that infect in nonsaline environments (30). Alternatively, it was also discussed whether the low adsorption rates of haloarchaeal viruses are due to differences in the surface structures of bacteria and archaea (29). However, the fast adsorption of HFTV1 now shows that slow binding of most haloarchaeal viruses cannot solely be attributed to the high salinity conditions, and other factors might play a role.

**TABLE 1:** Adsorption rates of different archaeal viruses and bacteriophages

Virus or bacteriophage	Host	Morphology	Adsorption rate (mL/min)	Source or reference
Euryarchaeal viruses				
HFTV1	Haloferax gibbonsii LR2-5	Icosahedral noncontractile tail	1.8 × 10^−9^	This study
HHTV-1	Haloarcula hispanica	Icosahedral noncontractile tail	2.9 × 10^−13^	29
His1	Haloarcula hispanica	Lemon shaped	1.9 × 10^−12^	51
His2	Haloarcula hispanica	Pleomorphic	5 × 10^−12^	52
HRPV9	*Halorubrum* sp. strain SS7-4	Pleomorphic	8.5 × 10^−11^	53
HHIV-2	Haloarcula hispanica	Icosahedral with internal membrane	3.7 × 10^−12^	23
HCIV-1	Haloarcula californiae	Icosahedral with internal membrane	5.7 × 10^−11^	28
Crenarchaeal viruses				
STIV1	Sulfolobus solfataricus	Icosahedral with membrane	2 × 10^−9^	54
SIRV2	Sulfolobus islandicus	Rod shaped	2 × 10^−8^	14
SMV1	Sulfolobus islandicus	Spindle shaped	7 × 10^−9^	55
SSV9	Sulfolobus islandicus	Spindle shaped	8.4 × 10^−11^	56
Bacteriophages				
T1	Escherichia coli	Icosahedral noncontractile tail	3 × 10^−9^	57
T2	Escherichia coli	Icosahedral noncontractile tail	2.1 × 10^−9^	58
φ6	Pseudomonas phaseolicola	Enveloped	3 × 10^−10^	59
PM2	Pseudoalteromonas espejiana	Icosahedral with internal membrane	2 × 10^−10^	60
SCTP-1	*Salicola* sp. strain PV3	Icosahedral noncontractile tail	3.4 × 10^−10^	29

We show that HFTV1 likely uses an S-layer glycoprotein as receptor. The composition of the archaeal cell envelope varies considerably between archaeal species. The sole structural cell wall components of haloarchaea and many crenarchaea are S-layer proteins. In the case of the well-studied crenarchaeon Sulfolobus acidocaldarius, the S-layer consists of two structurally distinct proteins, SlaA and SlaB, which together form an ordered 2-dimensional paracrystalline sheet around the cell (31). The membrane-proximal face of the S-layer consists of tripod-like SlaB trimers. The SlaB trimers support the outer canopy of the S-layer, which is formed by an array of tightly interwoven boomerang-shaped SlaA dimers (31). Deletion of genes encoding SlaB leads to mutant cells with partial S-layers consisting solely of SlaA. It was shown that SlaB-depleted strains were less susceptible to Sulfolobus spindle-shaped virus (SSV) infection, suggesting that SlaB is the receptor for this virus (32). In contrast, when SlaA is deleted, the S-layer does not assemble anymore, and cells are deformed. Thus, SlaA and SlaB both have different functions in the *Sulfolobus* S-layer.

The S-layer of H. volcanii consists of a hexagonal array of tightly interacting S-layer proteins that contain immunoglobulin-like domains (25). There is only one S-layer protein encoded, and it is lipid anchored in the cell membrane, in contrast to the S-layer protein of *Sulfolobus*, which is anchored by a transmembrane domain (33, 34). Interestingly, some haloarchaea, such as H. gibbonsii, encode two or more different S-layer proteins (in LR2-5, HfgLR_11210, and HfgLR_04635). These proteins in H. gibbonsii LR2-5 have a similar amino acid sequence and likely do not have such structurally dissimilar functions as the crenarchaeal SlaA and SlaB. It is not known if both LR2-5 genes are redundant, as their high sequence similarity might suggest. In addition, it is unclear if individual cells express only one of the genes or if S-layers in these organisms consist of a mixture of both proteins. At least for H. gibbonsii LR2-5, we have previously determined by mass spectrometry that both proteins are expressed (18). However, as we have used cell pellets for this analysis, we cannot detect S-layer differences at the single-cell level, and thus, there might be individual cells that only express one S-layer protein.

Deletion of *hfgLR_11210* results in viable H. gibbonsii LR2-5 cells, suggesting that a bona fide or partial S-layer is still being formed by the HfgLR_04635 protein, which can (partially) replace the function of HfgLR_11210. Deletion of *hfgLR_11210* results in the strain becoming resistant to HFTV1 infection, which shows that the S-layer protein (HfgLR_11210) encoded by H. gibbonsii LR2-5 is an essential receptor for HFTV1. As only *hfgLR_11210* is essential for HFTV1 infection, downregulation or deletion of this gene might even be a strategy of the host to escape viral infection. The multiple insertions that we observed in the gene *hfgLR_11210* could also indicate a mutational hot spot around the position encoding amino acids 237 to 246 in HfgLR_11210. Analysis of metagenome sequences of the original environment might give insight into this hypothesis.

Little is known about the receptors used by other haloviruses. The only other receptor of a haloarchaeal virus that has been identified previously is that of Halorubrum pleomorphic virus 6 (HRPV-6). This virus, like HFTV1, uses the S-layer of its host, *Halorubrum* sp. SS7-4, as receptor. Binding of HRPV-6 to the S-layer leads to activation of the viral fusion protein and results in virus-cell membrane fusion and genome delivery (12). Since HFTV1 is a tailed virus without a lipid envelope, viral fusion with the cell membrane is not the likely mechanism of entry for this virus.

As the S-layer is essential for adsorption, we assume that binding of the S-layer might be the first step in genome delivery. Analysis with electron microscopy showed that HFTV1 particles can bind to the surface both with their heads and with their tails. It is possible that the S-layer is the only receptor with which both parts of the virion interact or that head and tail interact with two distinct receptors. Adsorption where virions are orientated with their heads toward the cell envelope and their tails perpendicular to it seems unfavorable for genome delivery, which we assume would occur through the tail tube, as is the case for other dsDNA tailed viruses infecting bacteria. Indeed, most tailed dsDNA viruses bind with their tail tubes to the cell surface prior to genome delivery. However, some cases of virion head binding have also been observed, specifically in nonmodel viruses. For example, jumbo phages PTm1 and PTm5 infecting the bacterium Tenacibaculum maritimum have lytic life cycles with a latent period of 90 min (35). Viral particles have flexible fiber-like head appendages of 50 to 100 nm long. TEM observations on phage adsorption to the bacterial cell surface showed particles that seem to adsorb head fiber first on the cell during short incubation times (15 or 25 min), and the usual tail-first adsorption was observed later (35).

Such time-dependent reorientation of the particle is also observed for gene transfer agents (GTAs). These are bacteriophage-like genetic exchange elements that resemble small DNA bacteriophages and which transfer random pieces of the producer cell’s genome to recipient cells (36). The small phage-like particle RcGTA produced by Rhodobacter capsulatus looks like a tailed bacteriophage, and the capsid’s head is decorated by triangular spikes that are needed for binding to the capsule, which is a polysaccharide layer at the outside the bacterial cell envelope. RcGTA particles attach to cells in random orientations. In the model described for RcGTA-mediated DNA delivery, RcGTA particles attach to the cell surface by the head spikes, and the particle reorients by the binding of tail fibers to outer membrane receptors (37, 38). Next, the particle attaches to the membrane by putative receptor-binding domains of the baseplate, which is followed by penetration of the outer membrane (37, 38).

**FIG 6:**
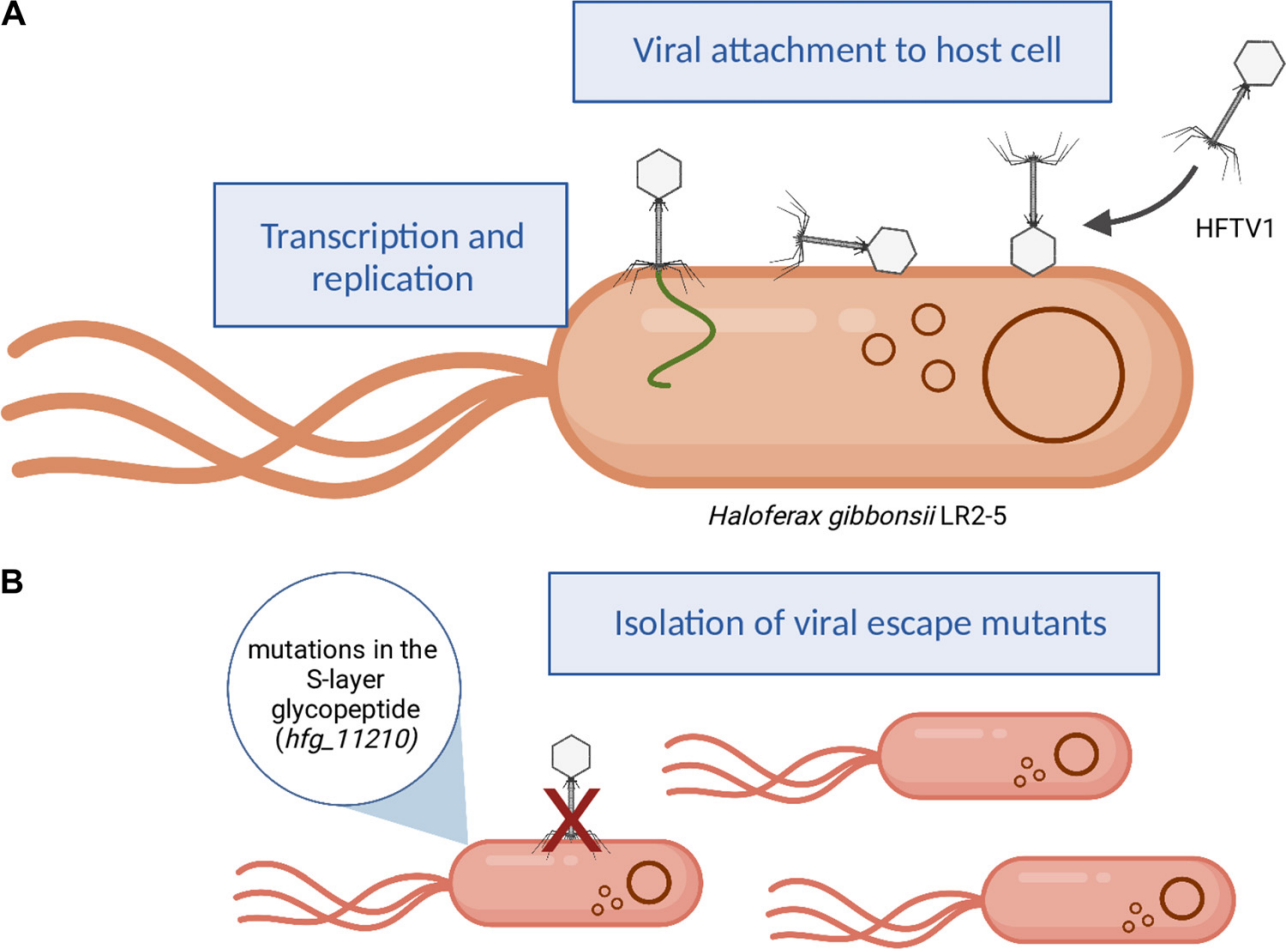
Model of HFTV1 binding to Haloferax gibbonsii LR2-5 cells. (A) HFTV1 adsorbs to the host cell within 3 min of infection. Viral particles attach to the host cell surface in random orientations (right side of the cell). For example, HFTV1 heads bind the cell, and the HFTV1 tails are perpendicular or parallel to the surface. Next, particles reorientate and attach to the S-layer by their tail (left). This is followed by viral genome (green) ejection and intracellular transcription and translation. Brown circles indicate the circular host genome and host megaplasmids. (B) Alterations in the S-layer glycopeptide Hfg_11210 of H. gibbonsii LR2-5 lead to escape mutants that are no longer susceptible to HFTV1. Figure created with BioRender.com.

We hypothesize that HFTV1 might also reorient the particle during the initial step of the entry in a time-dependent manner. We assume that the capsid head might contain specific structures, such as spikes or turrets, which have been observed in the heads of several bacteriophages (39–43) and have already been described for an haloarchaeal tailed HSTV-1 podovirus (family *Shortaselviridae*) (44). Indeed, specific turrets at the HFTV1 head might be visible (Fig. 4B). These structures might undergo specific and reversible interactions with the cell surface of H. gibbonsii LR2-5. A likely target might be the S-layer protein encoded by *HfgLR_11210*, but it might be equally likely that there is an interaction with glycans that also make part of the cell envelope. Next, the particle reorientates with help of the short tail fibers, which eventually leads to a specific and irreversible interaction of the base plate of HFTV1 with the S-layer protein. This event is followed by genome delivery, which we assume is by ejection of the viral dsDNA genome via the tail tube (Fig. 6).

This mode of binding to the host cell surface might increase the chances of successful genome delivery, as the orientation with which the viral particle first gets in contact with the cell surface is irrelevant for attachment to the cell. We assume that the head-first binding of HFTV1 might be responsible for the unusually fast adsorption rates measured for this virus in comparison with other haloarchaeal viruses. The actual genome delivery is not directly coupled with the adsorption, and it could still be possible that fast irreversible head-first binding is followed by a longer period of tail-first binding before genome delivery takes place. Future studies that include high-resolution microscopy techniques will be necessary to test this model.

In conclusion, although the overall morphology of HFTV1 is similar to other members of the class *Caudoviricetes*, its mechanism of adsorption to the cell is rather unusual and likely involves interaction with two distinct parts of the virion, both the head and tail. Interestingly, this mode of adsorption and host recognition is not unique to archaeal viruses and has also been observed for some bacteriophages and GTAs. This demonstrates that this mechanism might be conserved among diverse mobile genetic elements like viruses and GTAs of bacteria and archaea and more common than previously thought. This also highlights the importance of studying new model viruses in order to uncover the full diversity of microbial viral infection strategies.

## MATERIALS AND METHODS

### Virus and archaeal strain and their growth conditions.

Haloferax gibbonsii LR2-5 and HFTV1 (17) were cultured as described previously (45–47). For details, see Text S1 in the supplemental material.

### Plaque assay and preparation of virus stock.

For preparation of virus stock, semiconfluent plates were produced using the double-layer method (17, 18). Plaque and spot assays were performed as described previously (18). For details, see Text S1.

### HFTV1 stability.

The temperature stability of HFTV-1 was examined by incubating small aliquots (500 μL) of HFTV1 stocks at different temperatures ranging from 4°C to 100°C. After 1 h or 5 h of treatment in a thermoshaker, the infectivity was determined by plaque assay.

To test the effects of NaCl concentration on HFTV1 infectivity, virus stock was diluted 1:1,000 in 23% MGM medium that contained variable concentrations of NaCl (0 to 5 M) and a constant concentration of 113 mM MgCl_2_, 108 mM MgSO_4_, and 71 mM KCl (61 mM Tris-HCl, pH 7.5). After 2 h and 24 h of incubation at room temperature, the number of infective particles was determined with plaque and spot assays.

### Adsorption assay and constant calculation.

Haloferax gibbonsii LR2-5 cells from the mid-logarithmic growth phase were infected at a multiplicity of infection (MOI) of 10^−3^, and viral adsorption was monitored by plaque assay as described previously (23). For details, see Text S1.

### Receptor saturation assay.

H. gibbonsii LR2-5 was grown to an OD at 600 nm (OD_600_) of 0.65 to 0.85 (1 × 10^8^ to 6 × 10^8^ CFU/mL) and infected using MOIs from 0.001 to 400 at 37°C. At 30 min postinfection, cells were removed by centrifugation (15,000 × *g*, 2 min, 4°C), and the number of nonadsorbed particles in the supernatant was determined by plaque assay and compared to the amount of virus present in a cell-free control (MGM medium).

### Superinfection assay.

H. gibbonsii LR2-5 cells (OD_600_ of 0.6) were infected with HFTV1 using an MOI of 10 and incubated at 37°C (with shaking, 140 rpm) for 1.5 h. Cells were washed twice with 37°C warm 23% (wt/vol) MGM medium in two rounds of centrifugation (4,000 × *g*, 20 min, 30°C). Subsequently, infected cells were subject to a second round of infection using an MOI of 0.1. The number of unadsorbed viral particles in the supernatant was determined by plaque assay and compared to a cell-free control and cells that underwent only one round of infection at an MOI of 0.1.

### Infection assay and virus life cycle.

The life cycle of HFTV1 was investigated by infecting H. gibbonsii LR2-5 culture with a cell density of OD_550_ of 0.8 and ~8.5 × 10^8^ CFU/mL at mid-exponential growth phase using an MOI of 10. The turbidity of infected and uninfected culture was monitored at OD_550_. Samples were collected at several time points postinfection. Cells and cellular debris were removed by centrifugation (4,000 × *g*, 20 min, 4°C), and supernatants were analyzed by plaque assay.

### Production and purification of HFTV1 particles.

To purify HFTV1, H. gibbonsii LR2-5 was grown to mid-exponential phase to an OD_550_ of 1.2 and then infected at an MOI of 10. HFTV1 particles were produced in liquid culture by infecting logarithmically growing LR2-5 cells at an OD_550_ of 1.2 (1.9 × 10^9^ CFU/mL) using an MOI of 10. After the lysis, the cells were removed by centrifugation (10,800 × *g*, 30 min, 5°C). Alternatively, the viruses were purified from a virus stock. Viruses were precipitated with two-step polyethylene glycol (PEG)-NaCl precipitation as described previously (17). For details, see Text S1.

### Time-lapse microscopy.

Haloferax gibbonsii LR2-5 cells were grown in Casamino Acids (CA) medium containing 18% salt water (SW). Sample preparation and light microscopy were carried out in a similar fashion as described in reference 48. Cells were imaged on an agarose pad with nutrients at ×100 magnification using an Axio Observer.Z1 (Zeiss) inverted microscope equipped with a heated (45°C) XL-5 2000 incubator running ZEN software. Cells were recorded with 10- to 15-min time-lapse movies. Microscopy images were processed to analyze cell shapes using Fiji and the MicrobeJ plugin (49, 50). For details, see Text S1.

### Transmission electron microscopy.

Samples (5 μL) of 2× purified HFTV1 or H. gibbonsii LR2-5 cells from early exponential phase (OD_600_ of ~0.1) infected with purified HFTV1 (MOI of 150) were adsorbed onto Formvar carbon-coated copper grids for 1 min and stained with 2% (wt/vol) uranyl acetate for 20 s. Imaging was performed with a Hitachi 7800 TEM (120 kV) equipped with a LaB6 filament and coupled to an Emsis Xarosa complementary metal oxide semiconductor (CMOS) camera (Emsis GmbH, Muenster, Germany).

### Isolation and sequencing of HFTV1-resistant mutants of H. gibbonsii LR2-5.

Resistant mutants were selected from H. gibbonsii LR2-5 liquid cultures after infection with HFTV1. Cells from mid-log phase (OD_600_ of 0.65) were infected at an MOI of 10, and cultures were incubated at 37°C for 24 h. The lysate was plated in different dilutions on 20% MGM plates and incubated at 37°C for 7 days to isolated individual resistant mutants. Single colonies were purified by streaking on a new plate three times. Resistance to HFTV1 was confirmed by spot-on-lawn assays as described previously (18).

### Annotation of genomic variants in escape mutants.

DNA was extracted as described previously (18) and subjected to Illumina sequencing. Reads were mapped to the H. gibbonsii LR2-5 genome, and single and small nucleotide variants were identified. For details, see Text S1.

### Data availability.

The three Illumina sequencing libraries supporting the findings of this study are openly available from the European Nucleotide Archive (study, accession no. PRJEB53889; samples, accession nos. ERS12284977, ERS12284978, and ERS12284979).
