# Supplementary figures and images for: Archaeal Host Cell Recognition and Viral Binding of HFTV1 to Its Haloferax Host
Source: mBio. 2023 Jan 19;14(1):e01833-22. doi: 10.1128/mbio.01833-22 (PMC9973310; doi:10.1128/mbio.01833-22)

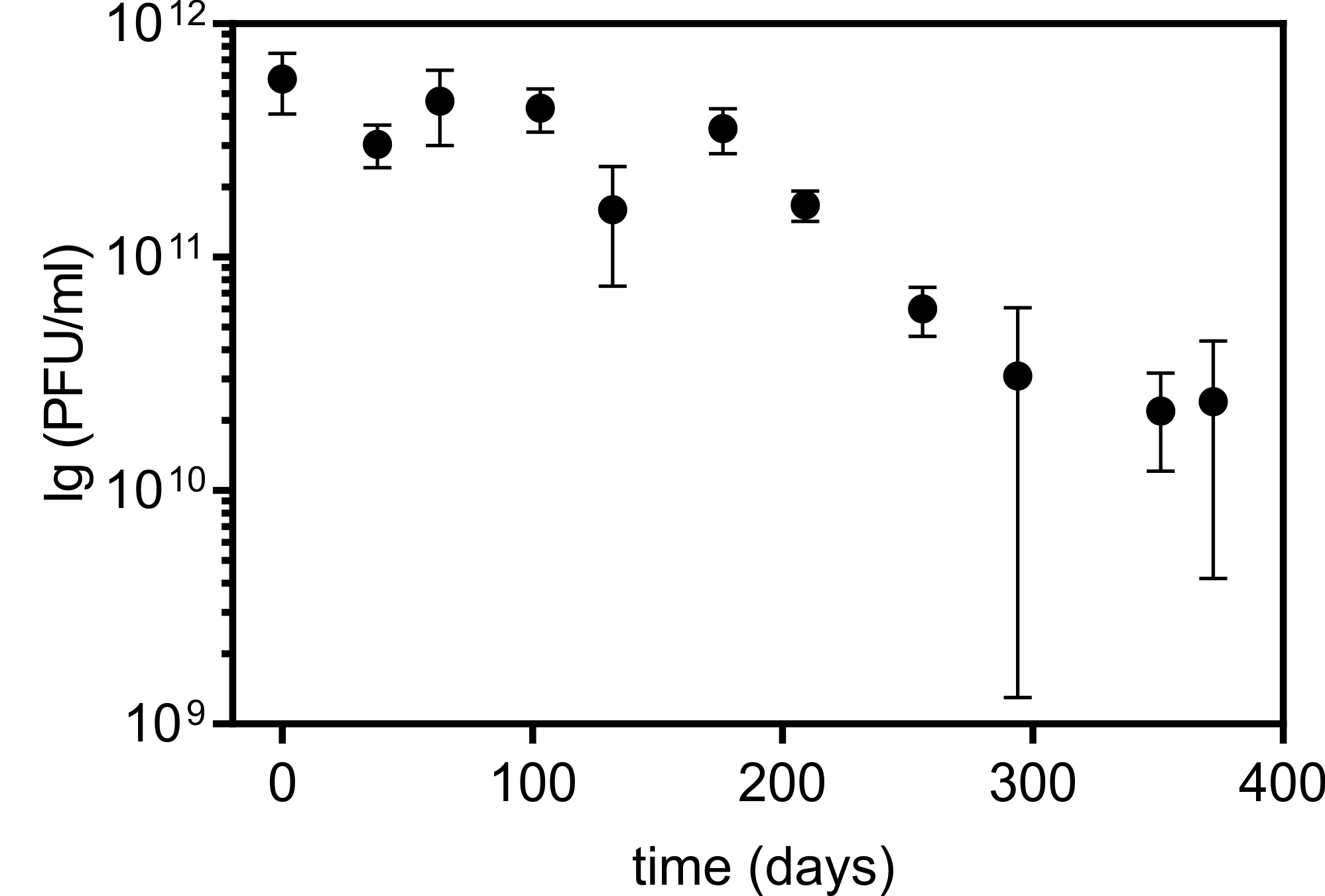

Supplement: FIG S1 [file mbio.01833-22-s0004.tif]

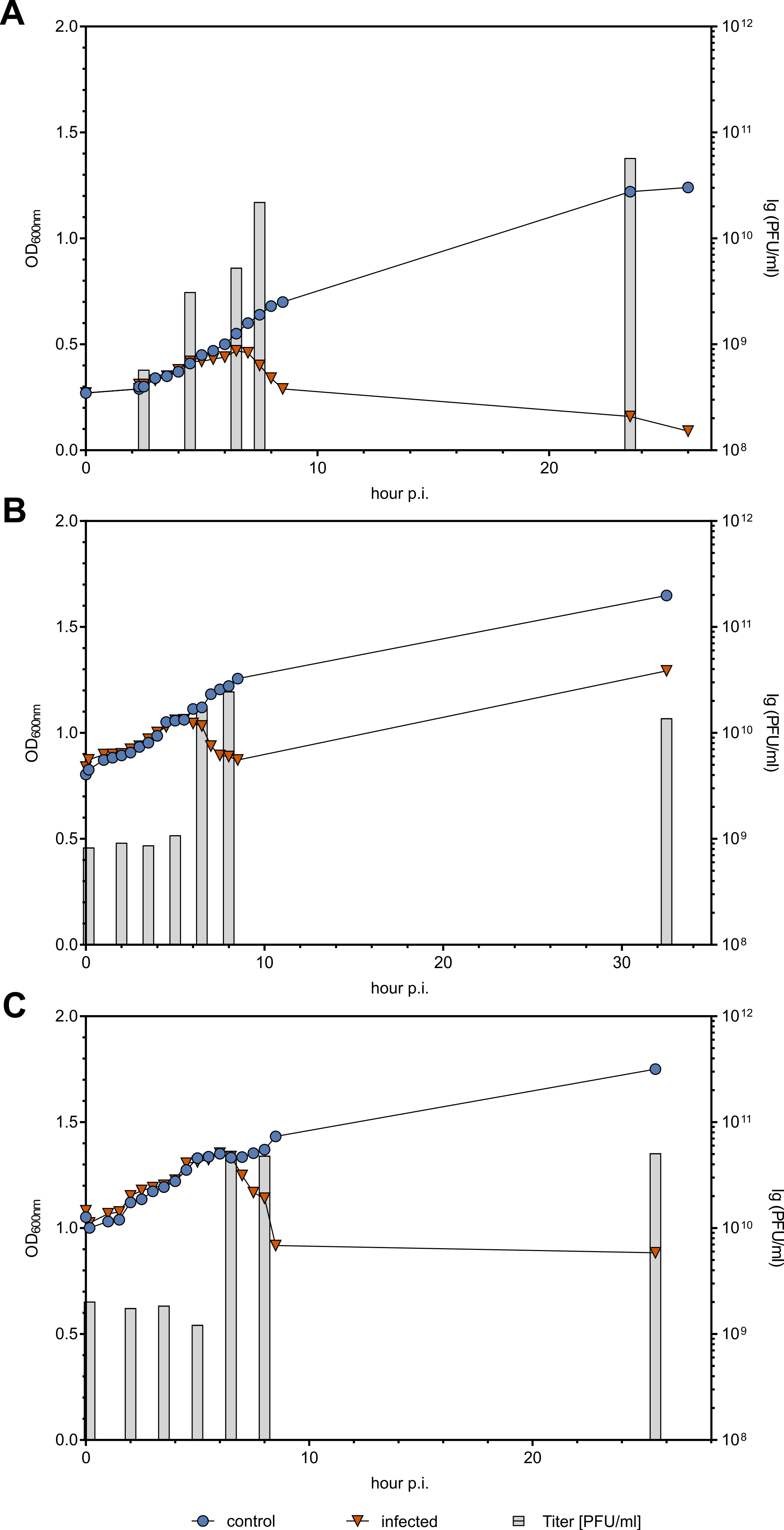

Supplement: FIG S2 [file mbio.01833-22-s0005.tif]

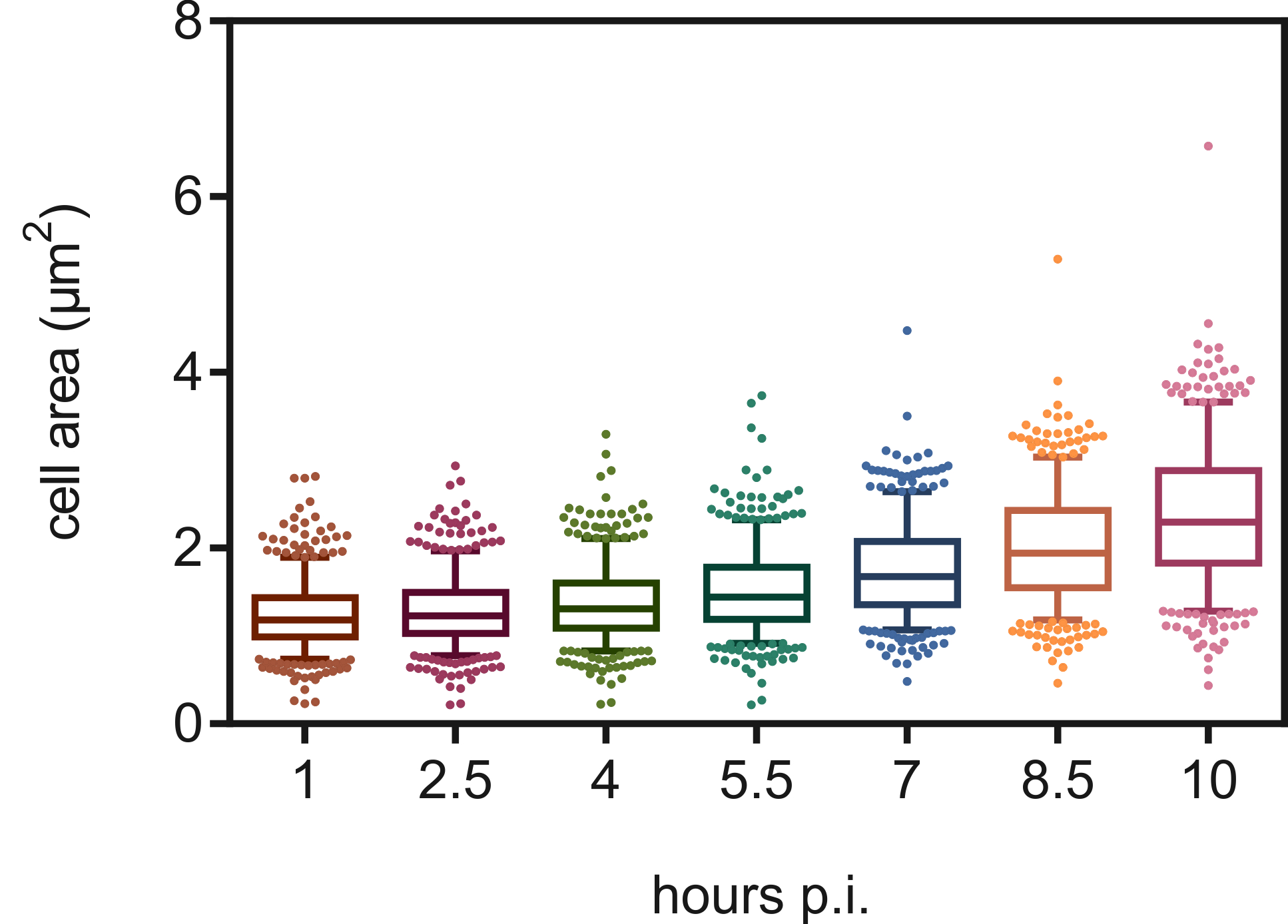

Supplement: FIG S3 [file mbio.01833-22-s0006.tif]

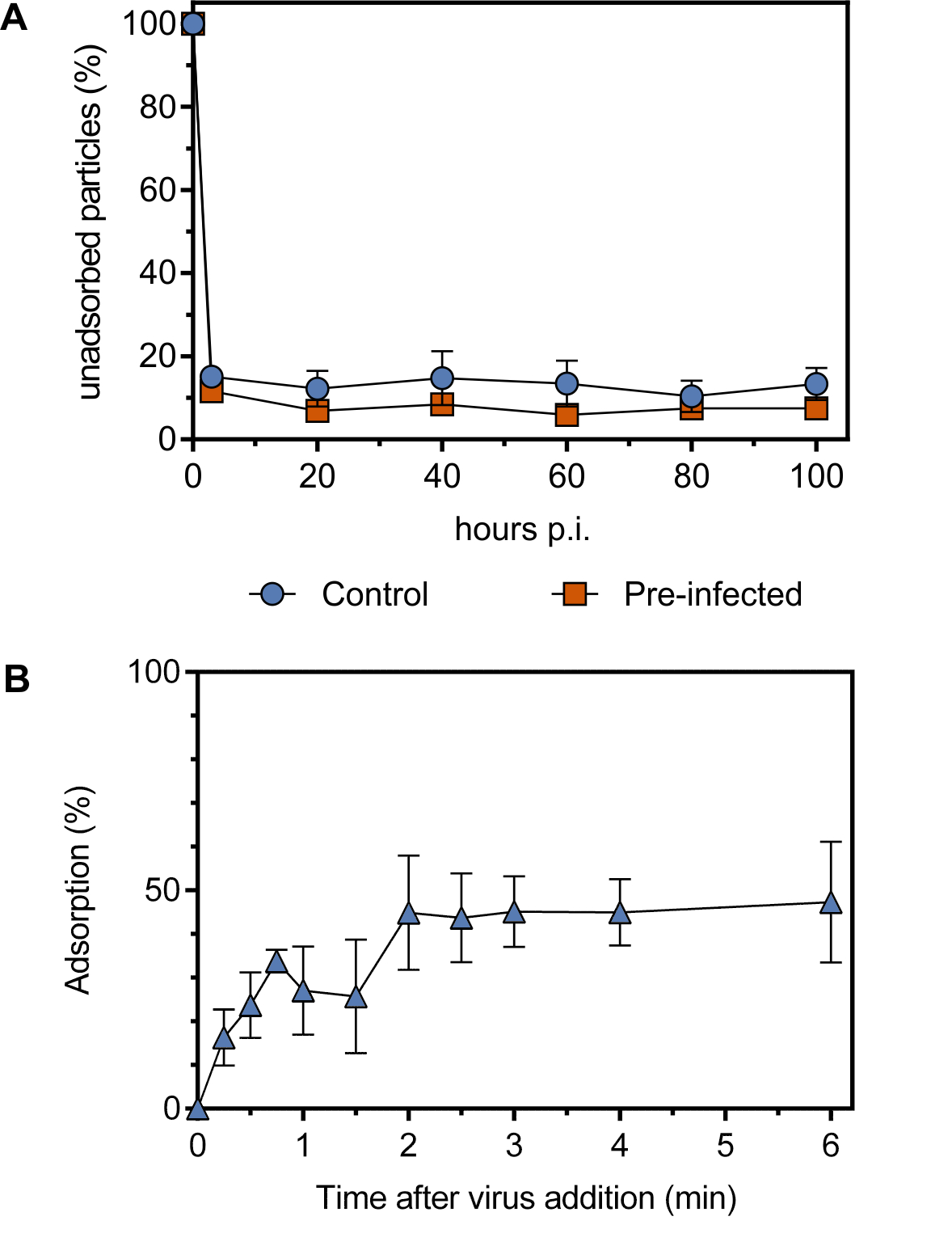

Supplement: FIG S7 [file mbio.01833-22-s0010.tif]

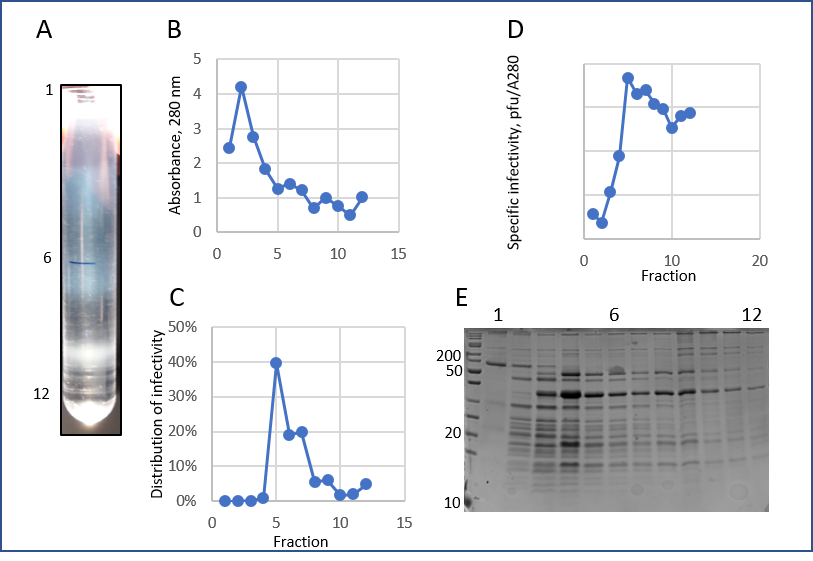

Supplement: FIG S4 [file mbio.01833-22-s0007.tif]

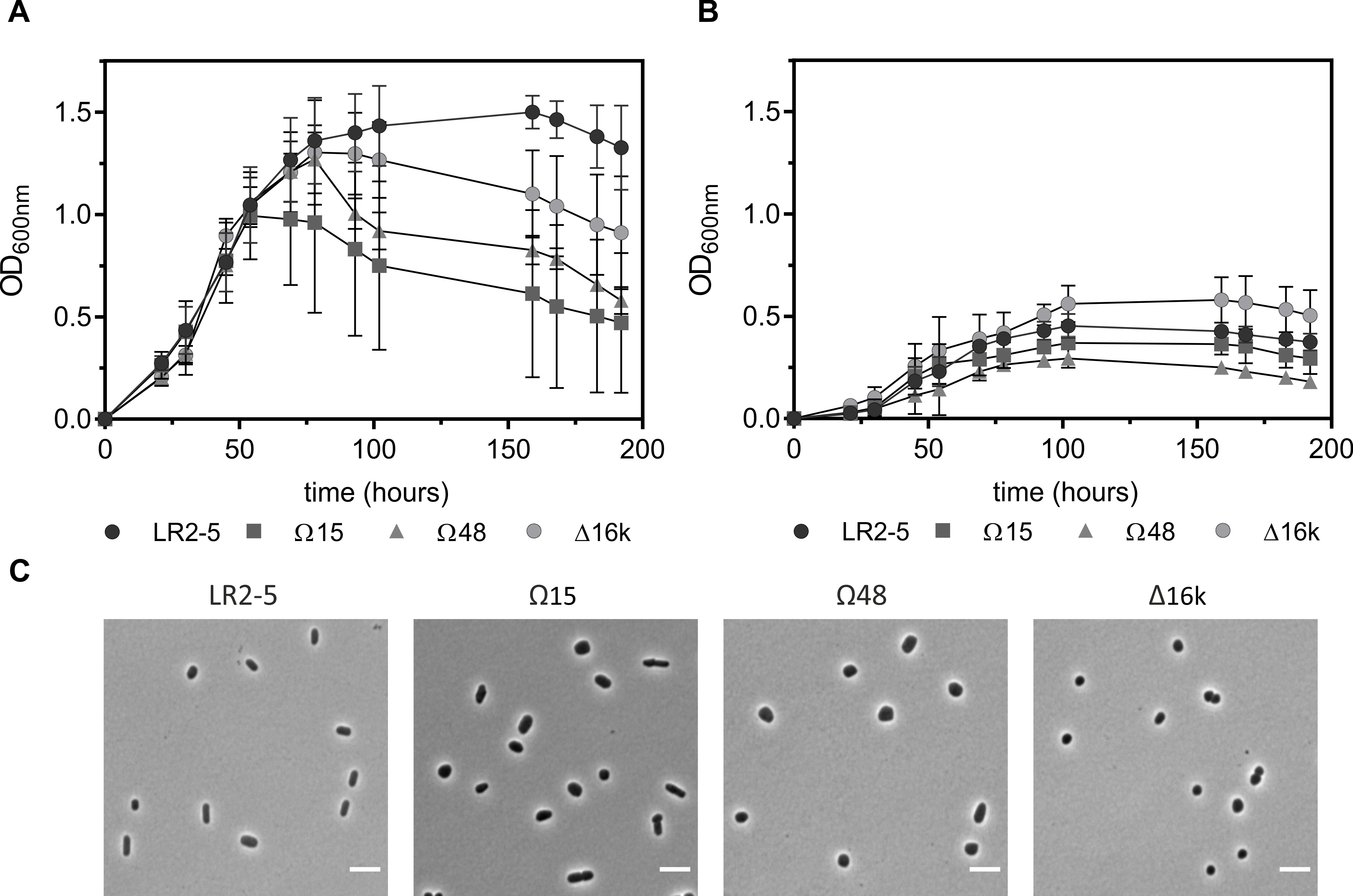

Supplement: FIG S5 [file mbio.01833-22-s0008.tif]

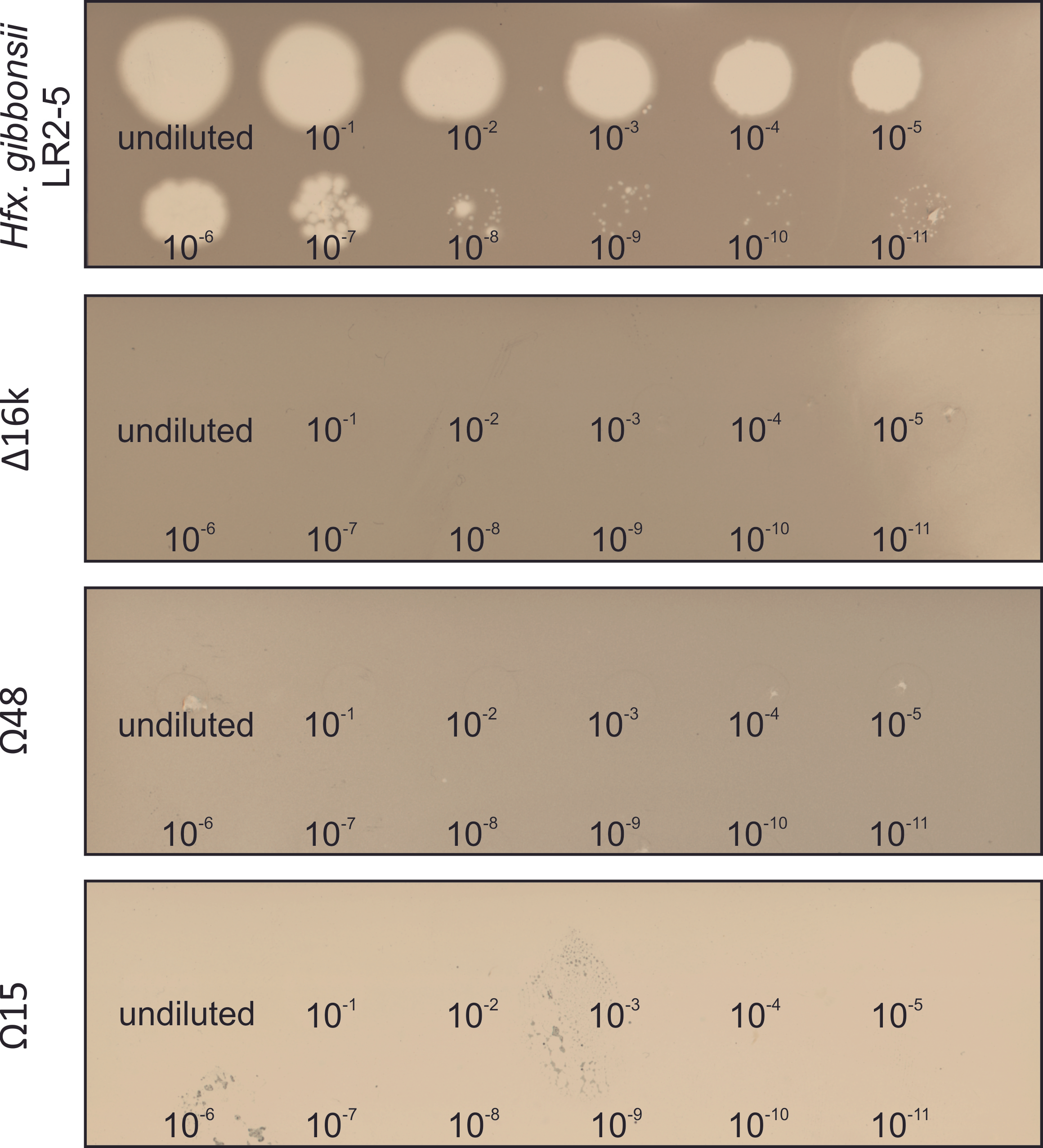

Supplement: FIG S6 [file mbio.01833-22-s0009.tif]
