# Supplementary material for: Archaeal Host Cell Recognition and Viral Binding of HFTV1 to Its Haloferax Host
Source: mBio. 2023 Jan 19;14(1):e01833-22. doi: 10.1128/mbio.01833-22 (PMC9973310; doi:10.1128/mbio.01833-22)
Supplement: TEXT S1 [file mbio.01833-22-s0001.docx]

## Supplemental Information to

## Archaeal host cell recognition and viral binding of HFTV1 to its Haloferax host

Sabine Schwarzer ^1,4^, Thomas Hackl ^2^, Hanna M. Oksanen ^3*^, Tessa E.F. Quax ^1,4*^

^1^ Archaeal virus-host interactions, Faculty of Biology, University of Freiburg, Schaenzlestrasse 1, 79104 Freiburg, Germany; sabine.schwarzer@biologie.uni-freiburg.de (T.E.F.Q. and S.S.)

^2^ Groningen Institute for Evolutionary Life Sciences, University of Groningen, 9747 AG Groningen, The Netherlands; t.hackl@rug.nl (T.H.).

^3^ Molecular and Integrative Biosciences Research Programme, Faculty of Biological and Environmental Sciences, University of Helsinki, Viikinkaari 9, 00014 Helsinki, Finland; hanna.oksanen@helsinki.fi (H.M.O.).

^4^ Biology of Archaea and Viruses, Groningen Biomolecular Sciences and Biotechnology Institute, University of Groningen, 9747 AG Groningen, The Netherlands; s.schwarzer@rug.nl (S.S.), t.e.f.quax@rug.nl (T.E.F.Q. and S.S.).

* Correspondence: hanna.oksanen@helsinki.fi (H.M.O.); t.e.f.quax@rug.nl (T.E.F.Q.)

## Supplemental Text

### Virus and archaeal strain and their growth conditions

*Haloferax gibbonsii* LR2-5 and HFTV1 [1] were aerobically grown at 37°C in modified growth medium (MGM) [2] containing artificial salt water (SW) [2], [3]. A 30% (v/v) stock of SW was produced and diluted to make the working media. Broth, solid agar, and soft-agar media contained 23%, 20%, and 18% (w/v) SW, yeast extract (0.1% w/v Oxoid) and peptone (0.5% w/v Oxoid), respectively. Agar (Bacto-Agar) was added to prepare solid (14 g/liter) or top-layer (4 g/Liter) media. *Hfx. gibbonsii* cells used for electron microscopy experiments were grown in selective CA (casamino acids) medium [4] prepared with 18% (w/v) SW buffered with 10 mM HEPES (4-(2-hydroxyethyl)-1-piperazineethanesulfonic acid) (pH 7.0) and supplemented with casamino acids (Difco) to a final concentration of 0.5% (w/v).

### Plaque assay and preparation of virus stock

An appropriate dilution series of HFTV1 stock was prepared using test tubes containing 5 mL of MGM broth. The virus was spread with its host cells using the double layer method. Dense host culture (300 µL) was mixed with 100 µL of appropriate virus dilution and 3 mL of melted soft agar and spread over a plate. Control plates with top agar and host cells, without the virus were included. The experiment was performed by using parallel plates. Plates were incubated for 2-3 days at 37°C and the number of infectious viruses i.e. the titer (PFU/mL) was determined.

For spot assay (titration), a mixture of host culture and melted soft agar was pour on a plate and the lawn was solidified and dried at room temperature for approximately 30 minutes. Virus dilutions (10 µL) were pipetted on the solid lawn. A control spot of medium only was included on each plate. The virus drops were dried for 30 minutes at room temperature prior to incubation for 4 days at 37°C.

For virus stock preparation, semi-confluent plates were produced by plaque assay. The top-layer agar was collected and 2 mL MGM medium was added per a collected plate. The lysate was incubated with aeration for 1.5 h at 37°C. HFTV1 particles were harvested by centrifugation (4,000 × g, 20 min, 4°C) and stored until use at 4°C.

### Time-laps microscopy

For imaging, cultures were diluted to an OD_600_ of 0.1 and 5 µl cell suspension was spotted on an agarose pad containing nutrients (0.3% [w/v] agar, 18% SW supplemented with casamino acids). After the sample was dried, the pad was flipped up-side down into a Delta T Dish (Bioptechs Inc.) and the dish was closed with a lid to avoid evaporation. The cells were observed at 100x magnification in the Phase contrast mode (PH3) using an Axio Observer.Z1 inverted microscope (Zeiss). The imaging procedure was carried out at 45°C over-night for 16.5 h with image acquisition every 10 or 15 min.

The FIJI/ImageJ plugin MicrobeJ was used to calculate the surface area of the cells from phase-contrast images taken at different time points after infection. MicrobeJ automatically detects and evaluates the particles in the image. The Plugin allows constraining the attributes in the user interface in order to exclude certain particles (dividing or lysed cells) or include missed particles upon reanalysis. Particles with an area outside 0.3 µm^2^ in the field were rejected. With this procedure, the cell area of HFTV1 infected *Hfx. gibbonsii* LR2-5 cells was determined at the indicated times and shown in a boxplot diagram in Figure S3.

### Adsorption assay

*Haloferax gibbonsii* LR2-5 cells (optical density at 600 nm [OD_600_] = 1.0; 2 x 10^9^ CFU/ml) were infected at a multiplicity of infection (MOI) of 10^-3^ and incubated aerobically at 37°C. HFTV1 infection was monitored in 50 mL reaction vessels. Samples were taken at 0.25, 1, and 2 min intervals and adsorption was stopped by diluting the samples 1:100 in ice-cold broth. The cells were removed by centrifugation, and the reduction in the number of PFUs in the supernatant was determined by plaque assay using *Hfx. gibbonsii* LR2-5 as a host. The adsorption rate constant was calculated as described previously using the formula, k = 2.3/Bt × log10(P_0_/P) [5] where B represents the concentration of host cells, P_0_ represents the concentration of free viruses at time point zero, and P represents the concentration of free viruses at the end of the experiment after a period of time t.

### Purification of HFTV1 particles

First, 4% (w/v) PEG 6000 (no NaCl added due to the high salinity of the medium) was dissolved by magnetic stirring for 1 h at 4 °C. After centrifugation (10,800 × g, 40 min, 5° C), more PEG was added to the supernatant to obtain a final concentration of 11 % (w/v). After dissolution of PEG and centrifugation (as above), the virus precipitate was dissolved in 18% SW buffer (around 50 times concentrated compared to the original growth volume). The aggregates were removed by centrifugation (6,300 × g, 10 min, 5° C). The viruses were purified by rate zonal centrifugation in 10-40 % (w/v) sucrose gradient in Sorvall TH641 rotor (18% SW buffer, 210,000 × g, 1.5 h, 15° C). After centrifugation, the gradient fractions (12 1-mL fractions) were analysed for their infectivity, absorbance (A_280_), and purity by sodium dodecyl sulfate–polyacrylamide gel electrophoresis (SDS-PAGE). For more pure particles, rate zonal centrifugation was followed by equilibrium (CsCl ρ=1.45 g/mL in 18% SW) and differential centrifugation as described previously [1] (114,000 × g, 2 h, 5 °C). Viruses were resuspended in 18% SW buffer.

Protein concentrations were determined by Bradford assay [6] using bovine serum albumin as a control. Specific infectivity (pfu/ mg of protein) was calculated as the ratio of titer to concentration. Proteins were analysed by tricine-SDS-PAGE [7] using 14% and 4 % (w/v) acrylamide in separation and stacking gels, respectively.

### Annotation of genomic variants in escape mutants

To obtain genomic DNA of escape mutants, cells of *Hfx. gibbonsii* LR2-5 were grown aerobically at 37^o^C with shaking (120 rpm) to an OD_600_ of 1.2 in MGM were collected by centrifugation (30 min at 5,000 g, room temperature) and processed for DNA extraction by Eurofins NGS Lab Constance (Constance). Cells were resuspended in ST buffer (1 M NaCl, 20 mM Tris-HCl, pH 7.5). Lysis solution (100 mM EDTA pH 8.0, 0.2 % SDS) was then added to the cells and 1 mL ethanol was layered on top of the aqueous solution to form two phases. DNA was spooled onto a capillary at the interface. The DNA was washed in ethanol and resuspended in 500 µl TE buffer (10 mM Tris-HCl pH 8.0, 10 mM EDTA). DNA was precipitated by adding 50 µl 3 M sodium acetate (pH 5.2) and 400 µL isopropanol and collected by centrifugation (16,000 g, 5 min). The pellet was washed with 70% ethanol and dried and the DNA was resuspended in 100 µl TE buffer. The sample was mixed with RNAse A (1 µL of 30 mg/mL in 50 % glycerol, Sigma-Aldrich Cat. R 4642: 45°C for ≥1 hour). The DNA was left at 4°C overnight for resuspension. Three 2x150 bp genomic libraries were prepared at Eurofins and sequenced on a NovaSeq 6000 platform.

To assess if the resistant clones are escape mutants, we compared sequencing data from the clones to the *Hfx. gibbonsii* reference. We mapped the Illumina reads with minimap2 [8] in short-read mode onto the reference genome for *Hfx. gibbonsii* strain LR2-5 and HFTV1 (NCBI accessions: CP06320[5-8] and MG550112). Single and small nucleotide variants were called with freebayes (`-p 1 -0 -F 0.2`) [9]. After filtering out low quality and low frequency loci, no
significant variants were detected. The large deletion in mutant Δ16k was identified from a complete lack in coverage in the sequencing depth profile and the small insertions in Ω15 and Ω48 (not detected by freebayes due to size) were identified visually in IGV [10] at the same locus as the deletion in Δ16k. We confirmed the insertions by local reassembly with SPAdes [11] and gene-reannotation with prodigal. Changes on the protein-level in Ω15 and Ω48 were analyzed based on multiple sequence alignments generated with MAFFT [12] and visualized with AliView [13]. The residual presence of viral DNA in the samples was analyzed by comparing the coverages across host sequences and the viral genome.

## References

[1] C. M. Mizuno *et al.*, “Novel haloarchaeal viruses from Lake Retba infecting Haloferax and Halorubrum species,” *Environ. Microbiol.*, vol. 21, no. 6, pp. 2129–2147, Jun. 2019, doi: 10.1111/1462-2920.14604.

[2] S. D. Nuttall and M. L. D. Smith, “HF1 and HF2: Novel bacteriophages of halophilic archaea,” *Virology*, vol. 197, no. 2, pp. 678–684, 1993, doi: 10.1006/viro.1993.1643.

[3] M. Dyall-Smith, “The halohandbook: Protocols for haloarchaeal genetics,” *Halohandb. Protoc. haloarchaeal Genet.*, no. March, pp. 1–144, 2009, Accessed: May 08, 2020. [Online]. Available: http://scholar.google.com/scholar?hl=en&btnG=Search&q=intitle:The+Halohandbook#2.

[4] T. Allers, H. P. Ngo, M. Mevarech, and R. G. Lloyd, “Development of Additional Selectable Markers for the Halophilic Archaeon Haloferax volcanii Based on the leuB and trpA Genes,” *Appl. Environ. Microbiol.*, vol. 70, no. 2, pp. 943–953, Feb. 2004, doi: 10.1128/AEM.70.2.943-953.2004.

[5] M. H. Adams, “Bacteriophages.,” *Bacteriophages.*, 1959.

[6] M. Bradford, “A Rapid and Sensitive Method for the Quantitation of Microgram Quantities of Protein Utilizing the Principle of Protein-Dye Binding,” *Anal. Biochem.*, vol. 72, no. 1–2, pp. 248–254, May 1976, doi: 10.1006/abio.1976.9999.

[7] H. Schägger and G. von Jagow, “Tricine-sodium dodecyl sulfate-polyacrylamide gel electrophoresis for the separation of proteins in the range from 1 to 100 kDa,” *Anal. Biochem.*, vol. 166, no. 2, pp. 368–379, Nov. 1987, doi: 10.1016/0003-2697(87)90587-2.

[8] H. Li, “Minimap2: pairwise alignment for nucleotide sequences,” *Bioinformatics*, vol. 34, no. 18, pp. 3094–3100, Sep. 2018, doi: 10.1093/bioinformatics/bty191.

[9] E. Garrison and G. Marth, “Haplotype-based variant detection from short-read sequencing,” Jul. 2012, Accessed: Jun. 22, 2022. [Online]. Available: http://arxiv.org/abs/1207.3907.

[10] H. Thorvaldsdottir, J. T. Robinson, and J. P. Mesirov, “Integrative Genomics Viewer (IGV): high-performance genomics data visualization and exploration,” *Brief. Bioinform.*, vol. 14, no. 2, pp. 178–192, Mar. 2013, doi: 10.1093/bib/bbs017.

[11] A. Bankevich *et al.*, “SPAdes: A new genome assembly algorithm and its applications to single-cell sequencing,” *J. Comput. Biol.*, vol. 19, no. 5, pp. 455–477, May 2012, doi: 10.1089/cmb.2012.0021.

[12] T. Nakamura, K. D. Yamada, K. Tomii, and K. Katoh, “Parallelization of MAFFT for large-scale multiple sequence alignments,” *Bioinformatics*, vol. 34, no. 14, pp. 2490–2492, Jul. 2018, doi: 10.1093/bioinformatics/bty121.

[13] A. Larsson, “AliView: a fast and lightweight alignment viewer and editor for large datasets,” *Bioinformatics*, vol. 30, no. 22, pp. 3276–3278, Nov. 2014, doi: 10.1093/bioinformatics/btu531.

**Supplemental Tables**

**Table S1: Sequence read counts from Illumina Sequencing of *Haloferax gibbonsii* LR2-5 escape mutants.**

|  |  |  |  | Sequence | Sequence length | Mean coverage | Median coverage |
| --- | --- | --- | --- | --- | --- | --- | --- |
| Ω48 | | |  | CP063205.1 | 2999641 | 216.43 | 217 |
|  |  |  |  | CP063206.1 | 608598 | 207.2 | 207 |
|  |  |  |  | CP063207.1 | 322970 | 205.88 | 206 |
|  |  |  |  | CP063208.1 | 65035 | 303.81 | 303 |
|  |  |  |  | MG550112.1 | 38059 | 7944.29 | 7948 |
| Δ16k | | |  | CP063205.1 | 2999641 | 372.95 | 375 |
|  |  |  |  | CP063206.1 | 608598 | 356.58 | 358 |
|  |  |  |  | CP063207.1 | 322970 | 318.6 | 321 |
|  |  |  |  | CP063208.1 | 65035 | 634.89 | 635 |
|  |  |  |  | MG550112.1 | 38059 | 1.54 | 1 |
| Ω15 | | |  | CP063205.1 | 2999641 | 440.16 | 445 |
|  |  |  |  | CP063206.1 | 608598 | 448.64 | 452 |
|  |  |  |  | CP063207.1 | 322970 | 397.4 | 401 |
|  |  |  |  | CP063208.1 | 65035 | 671.6 | 673 |
|  |  |  |  | MG550112.1 | 38059 | 7895.13 | 7918 |

## Supplemental Figures

**Supplementary Figure S1: HFTV1 is stable for at least 6 months at 4°C.** The infectivity of HFTV1 agar stock was monitored over a 12-month period. The stock was stored at 4°C and the titer (pfu/ml) was determined by plaque assay. Scale bars represent two technical replicates.

**Supplementary Figure S2: Single-step growth curves of HFTV1**. *Hfx. gibbonsii* LR2-5 cells were grown to logarithmic phase and infected with an MOI of 10 at 37°C. (A) Cultures infected at OD_600_ of 0.3 (~1.0 × 10^7^ CFU/mL), (B) OD_600_ of 0.8 (~2.0 × 10^9^ CFU/mL), and (C) OD_600_ of (~4.0 × 10^9^ CFU/mL). To remove unadsorbed viral particles the cells were washed thoroughly 20 min post infection (p.i.) and transferred to fresh medium at 37°C. The growth curves of infected (triangles) and uninfected (circles) cultures and the number of free viruses (pfu/ml) are shown.

**Supplementary Figure S3: Cell size increase during time-course of infection.** Box plots showing cell area distributions of *Hfx. gibbonsii* LR2-5 cells infected with HFTV1. Boxes represent values from >600 cells analyzed at 1-10 h p.i. (1.5 h intervals). After 10 hours p.i. cell area increased ~1.5 fold. A line within the box marks the median. The upper boundary represents the 75^th^ percentile. Lower whisker represents minimum values and the upper whisker represents maximum values.

**Supplementary Figure S4: Improved purification of HFTV1 particles by rate zonal centrifugation in sucrose.** (A) Polyethylene glycol-NaCl precipitated HFTV1 particles were separated in a linear 10-40% (w/v) sucrose gradient (18% SW buffer; 210 000 ×g, 1.5 h, 15 °C; Sorvall TH641 rotor) and fractionated into 12 fractions (numbering shown on left). Fractions were analysed by their (B) absorbance at 280 nm, (C) infectivity (total pfus), (D) specific infectivity (pfu/A_280_), and (E) protein content by Coomassie blue stained SDS-PAGE gel. Fraction numbers are shown on top. Mass marker (kDa) is on left.

**Supplementary Figure S5: Growth of *Hfx. gibbonsii* LR2-5 and its escape mutants in liquid culture.** (A) Typical Growth curve of *Hfx. gibbonsii* LR2-5, Ω15, Ω48, and ∆16k in MGM medium with 18% SW. The average optical density was calculated from three independent technical replicates. Error bars represent the standard deviation. (B) Typical Growth curve of *Hfx. gibbonsii* LR2-5, Ω15, Ω48, and ∆16k in CA medium with 18% SW. (C) Phase contrast light microscopy images show the cell shapes of *Hfx. gibbonsii* LR2-5 (OD_600_ 0.45) and its escape mutants Ω15 (OD_600_ 0.44), Ω48 (OD_600_ 0.26), and ∆16k (OD_600_ 0.31) at mid-exponential growth phases.

**Supplementary Figure S6: Susceptibility of *Haloferax gibbonsii* LR2-5 and escape mutants to HFTV1.** Spot-on-lawn assay conducted with lawns of *Hfx. gibbonsii* LR2-5, Δ16K, Ω48, and Ω15. Different dilutions of HFTV1 lysate (undiluted 3 × 10^11^ pfu/ml) were spotted on the respective host lawns and incubated for 4 days. Clearing of the cellular lawn appeared only on *Hfx. gibbonsii* LR2-5 lawns, whereas no indication of cell lysis was observed when HFTV1 lysate was spotted on the host lawns of the escape mutants Δ16K, Ω48, and Ω15.

**Supplementary Figure S7: Superinfection exclusion assay.** *Hfx. gibbonsii* LR2-5 cells were infected with an MOI of 10 and incubated for 1.5 hours at 37°C. Unbound particles were removed by washing and the cells were subjected to a second round of infection with HFTV1 at an MOI of 0.1. Blue circles show the adsorption kinetics of particles to uninfected control-cells. Orange squares represent the adsorption to cells pre-infected with HFTV1. Error bars represent the standard deviation of three independent experiments. If the error bars are not visible, the deviation could not be resolved graphically.

**Supplementary Figure S8: Adsorption efficiency of HFTV1 to the Δ16k escape mutant of *Hfx. gibbonsii* LR2-5.** (A) The adsorption efficiency of HFTV1 to the Δ16k escape mutant of LR2-5 was determined using cells at the mid logarithmic growth phase (OD_600_ 1.0; ~2 × 10^9^ CFU/ml) which were infected with HFTV1 using an MOI of 0.001 at 37°C. The number of unbound virus particles was determined at 0 – 6 minutes post infection by plaque assay. Error bars represent standard deviation from three experiments.

## Supplemental Movies

**Supplementary Movie S1: Phase contrast time-laps imaging of *Haloferax gibbonsii* LR2-5 infected with HFTV1.** Cells from early log growth phase (OD_600_ 0.2) were infected with HFTV1 at an MOI of 10. Cells were grown on an agarose nutrition pad in a thermomicroscope at 45°C and imaged every 10 minutes. Time post infection is indicated in hours.

**Supplementary Movie S2: Phase contrast time-laps imaging of non-infected *Haloferax gibbonsii* LR2-5 cells.** Cells were grown on an agarose nutrition pad in a thermomicroscope at 45°C and imaged every 15 minutes. Time post infection is indicated in hours.
